# Mitochondrial DNA heteroplasmy drives cortical neuronal disturbances in human organoids harbouring the common m.3243A>G mutation

**DOI:** 10.1038/s41467-026-74103-y

**Published:** 2026-06-21

**Authors:** Denisa Hathazi, Camilla Lyons, Daniel Lagos, Oliver Podmanicky, Mariana Zarate-Mendez, Yu Nie, Juliane S. Müller, Kieren S. J. Allinson, Huw Naylor, Majlinda Lako, Ibrahim Elsharkawi, Irena Muffels, Eva Morava, Tamas Kozicz, Patrick Chinnery, András Lakatos, Rita Horvath

**Affiliations:** 1 Department of Clinical Neurosciences, John van Geest Centre for Brain Repair, University of Cambridge, Cambridge, UK; 2 MRC Mitochondrial Biology Unit, University of Cambridge, Cambridge, UK; 3 Department of Histopathology, Cambridge University Hospital, Cambridge, UK; 4 Light Microscopy Core, Cancer Research Cambridge Institute, Cambridge, UK; 5 Biosciences Institute, Newcastle University, Newcastle, UK; 6 Department of Genetics and Genomic Sciences, Icahn School of Medicine at Mount Sinai, New York, USA; 7 Present Address: Dubowitz Neuromuscular Centre, University College London, and Great Ormond Street Hospital, London, UK

**Keywords:** Neuroscience, Genetics, Neurology, Molecular biology

## Abstract

Mitochondrial diseases frequently affect the brain leading to severe and disabling neurological symptoms. The heteroplasmic m.3243 A > G mutation in *MT-TL1*, encoding mt-tRNA^Leu^, is responsible for ~80% of mitochondrial encephalomyopathy, lactic acidosis, and stroke-like episodes (MELAS), which is one of the most characteristic mitochondrial syndromes, leading to disability and early death. There are no animal models harbouring this mutation to provide precise mechanistic insights informing therapeutic interventions. Here, we generate a human iPSC-derived cerebral organoid slice model that recapitulates cortical architecture and mitochondrial pathology. Using biological assays and single-cell RNA sequencing, we uncover heteroplasmy-dependent transcriptional shifts and changes in key cellular processes in cortical neurons. Organoids with high heteroplasmy show a predominant impairment of deep-layer neurons triggered by mitochondrial stress, leading to axonal degeneration and apoptosis, similar to brain autopsy of a MELAS patient. Our findings provide insights into the vulnerability of long-range projection neurons in mitochondrial diseases, advancing our understanding of disease mechanisms with a view to potential therapeutic strategies.

## Abstract (web summary)

Here authors use human iPSC-derived cerebral organoids to model the pathogenic m.3243 A > G mitochondrial mutation and reveal heteroplasmy-dependent, cell-type-specific vulnerability in human cortex neurons.

## Introduction

Mitochondrial diseases are clinically heterogeneous genetic conditions, caused by the defect of energy production through oxidative phosphorylation (OXPHOS)^1^. Neurological manifestations are common and severely disabling, given that neuronal function relies on ATP generated through OXPHOS^2^. The mechanisms by which mitochondrial mutations lead to neuronal defects and disease phenotypes are poorly understood, impeding the development of therapeutic strategies. Discoveries have been hampered by the complexity of human mitochondrial genetics and the lack of reliable animal and cellular models.

Studying the downstream effects of mitochondrial DNA (mtDNA) mutations has been particularly challenging due to the variable heteroplasmy levels that characterise human mitochondrial diseases, such as Mitochondrial Encephalomyopathy with Lactic Acidosis and Stroke-like Episodes (MELAS)^3,4^. While mouse models have been successfully developed to study nuclear-encoded mitochondrial disorders^5^, generating heteroplasmic mtDNA mutations in mice has proven difficult, with the few existing mtDNA mouse models failing to recapitulate the neurological pathologies observed in patients^3,4^. Recent advances in single-nucleotide mtDNA editing have enabled the generation of heteroplasmic variants in cellular and animal models^5–8^. However, these techniques do not allow for transgenic manipulation of all mtDNA mutations. Importantly, there are no animal models available for the heteroplasmic m.3243 A > G variant, one of the most common mtDNA variants^9^.

In fact, the m.3243 A > G mutation in the *MT-TL1* gene has been detected in almost half of adult patients with mitochondrial disease in clinical cohorts presenting with broad phenotypes of maternally inherited diabetes and deafness (MIDD), chronic progressive ophthalmoparesis (CPEO) and mitochondrial myopathy, often accompanied by disabling neurological symptoms, including ataxia, peripheral neuropathy and Stroke-like Episodes (SLEs)^10^. Thus, there is an unmet need for human-based research platforms to study its effects and develop targeted therapies.

Recent in vitro technologies enabled studies using human neuronal models harbouring the m.3243 A > G mutation^11^. A few studies involved human induced pluripotent stem cell (hiPSCs)-derived glutamatergic neurons with m.3243 A > G, including iNeurons^12,13^, motor neuron (MN) progenitors and MNs^14^, describing oxidative phosphorylation induced structural and metabolic disturbances. Distinct changes have also been described for other cell types, such as fibroblasts and hiPSCs carrying m.3243 A > G^11,13^. Therefore, how the varying degree of heteroplasmic mtDNA affects various somatic neural cells in a tissue environment is a pertinent question to address, which may shed light on key targetable downstream mechanisms.

Three-dimensional (3D) hiPSC-derived multicellular neural organoid models offer new promise for cell type-specific mechanistic discoveries in human neurological disease and treatment strategies^15,16^. Cortical organoid slice culture systems allow long-term assessments, perturbations and easy access for monitoring outcome measures in several neural cell types^17^. In addition, 3D organoids allow neurons to develop within a structured tissue-like environment, preserving key cell–cell interactions and spatial gradients essential for neuronal diversification and maturation^18,19^. Specifically, the presence of layer-specific cortical neuronal identities in organoids, including deep- and upper-layer subtypes, is an advantage over 2D monolayer culture models in which simultaneous divergence of neuronal subtypes seldom occurs^17,19^.

Human cortical organoid models have not yet been generated to uncover the role of m.3243 A > G heteroplasmy in orchestrating cellular pathology in various neuronal subtypes. To overcome this problem, we present a cortical organoid slice model comprising 756 slices generated from seven iPSC lines derived from two patients carrying the m.3243 A > G variant across five batches. We use a combination of single-cell transcriptomic approaches and cell cultures-based assays to reveal heteroplasmy-driven cellular changes, with a focus on neuronal subpopulations. We show that organoid slices display consistent tissue composition and stable heteroplasmy rates associated with mitochondrial dysfunction. Specifically, we provide direct evidence that high m.3243 A > G heteroplasmy leads to impaired bioenergetics, disturbed neuronal structure and function, associated with a significant loss of cortical deep-layer neuronal population in organoids, similar to the postmortem human adult brain of patients with MELAS.

## Results

### m.3243 A > G hiPSC-derived organoids have homogeneous cell populations and retain the mtDNA mutation

**Fig. 1:**
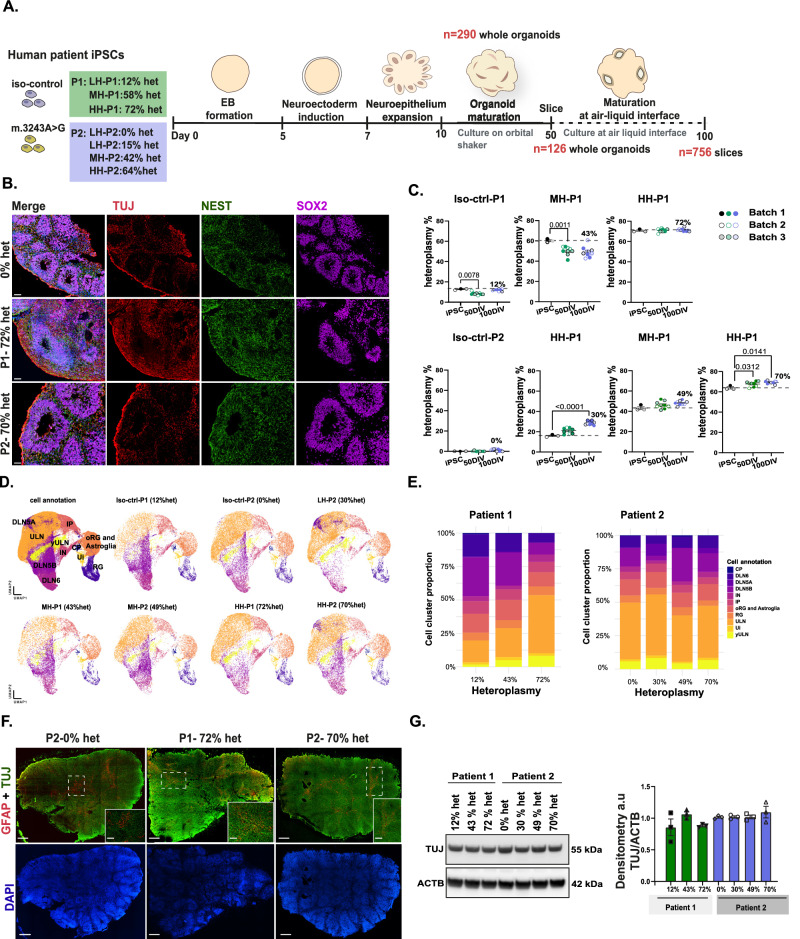
m.3243 A > G ALI-COs retain the mutation and exhibit consistent cellular populations. **A** Schematic representation organoid generation process and the human iPSC lines used in this study. **B** Representative immunofluorescence images of cortical organoids at DIV 30, showing SOX2⁺ progenitor cells (violet), surrounded by immature TUJ⁺ neurons (red) and NESTIN⁺ progenitor cells (green). Scale bar = 50 µm. **C** Heteroplasmy levels in the starting iPSCs and cortical organoids at DIV 50 and 100. For organoids, heteroplasmy was measured across three independent batches, with three organoids analysed per batch to account for intra- and inter-batch variability *n* = 3 for iPSCs and *n* = 9 for organoids. Data represent the mean ± SEM; *n* = *3* for iPSCs and *n* = *9* for organoids (three independent batches, three organoids per batch). A one-way mixed-effects analysis followed by Dunnett’s multiple comparisons test was applied (overall *p*-values indicated in the graphs). **D** Uniform manifold approximation and projection (UMAP) plots illustrating 11 colour-coded clusters identified in the scRNA-seq analysis, presented for the merged dataset and for each individual heteroplasmic organoid (*n*  =  119,591 cells). Clusters were defined using cell-marker genes and include the following populations: ULN, upper-layer cortical neurons; DLN5B and DLN5A, deep-layer neurons (layer 5, subtypes A and B); DLN6, deep-layer neurons (layer 6); IN, interneurons; yULN, young upper-layer neurons; IP, intermediate progenitors; RG, radial glia; oRG, outer radial glia; CP, choroid plexus; Ui, unidentified cells. **E** Bar plots depicting the cluster identity distributions, expressed as cell proportions within each pooled patient pair sample. **F** Representative immunofluorescence images of ALI-COs at DIV 100, showing TUJ⁺ (green) neurons and GFAP⁺ (red) glia across three biological replicates per group. Scale bar = 200 µm. **G** Representative WB images (left) of TUJ and ACTB protein expression in ALI-COs at DIV 100, along with quantification (right) of TUJ band intensity normalised to ACTB. Data represent the mean ± SEM; *n* = 3 independent ALI-COs per heteroplasmic line and isogenic controls from different batches. Statistical analysis: one-way ANOVA followed by Dunnett’s multiple comparison test (overall ANOVA *p*-values indicated in the graphs). Source data are provided as a Source Data file.

To assess the fidelity of human cortical organoids to recapitulate neural tissue architecture and the diversity of cell types harbouring the heteroplasmic m.3243 A > G variant, we employed a previously characterised long-term organoid slice culture platform^17,19^. We used iPSC lines derived from two patients carrying the m.3243 A > G mutation (Fig. 1A), clinically presenting with MIDD and pigmentary retinopathy (Patient 1) and MELAS, hearing loss and diabetes (Patient 2). The clinical data of these patients are summarised in Supplementary Table S1. From each line, we generated low, medium, and high heteroplasmy clones to model controlled heteroplasmy ranges independent of the original bulk heteroplasmy. These clonal lines were subsequently expanded in three ranges of heteroplasmy levels. For patient 1, we developed three clones with heteroplasmy levels of 12, 58, and 72%. For patient 2, we generated four iPSC clones with 0, 15, 42, and 64% heteroplasmy (Supplementary Fig. S1A). The clones with the lowest heteroplasmy − 12% for patient 1 and 0% for patient 2 − were below the mutational threshold and were used as isogenic controls. When assessed by phase contrast microscopy embryonic bud formation was comparable between all heteroplasmic organoids and their isogenic controls at 10 days in vitro (DIV) (Supplementary Fig. S1B). We performed immunohistochemical analyses to verify potential heteroplasmy variation-driven differences in tissue architecture in 30 DIV organoids. We observed the germinal zones in all heteroplasmy groups, with the presence of the typical early neuronal cell phenotypes, including SOX2^+^ neural epithelium, NEST^+^ progenitors, and TUJ^+^ immature neurons (Fig. 1B and Supplementary Fig. S1C). This consistent tissue organisation allowed us to set up organoid slice cultures, which can be cultivated long-term to quantitatively compare heteroplasmy-induced effects imposed on mature neurons. Organoids were sliced between 45–50 DIV and grown further until 100 DIV as air-liquid interface cortical organoid (ALI-CO) slices, as previously described^17,19^, which allows consistent cortical expansion and neuronal maturation. Normal development in mutant organoids mirrored normal brain development, and later symptom onset seen in patients with m.3243 A > G.

We then explored the cell type composition and maturity to uncover any biases that may affect the assessment of heteroplasmy levels and metabolic function across the different groups of organoids. Since heteroplasmy can change over time, we quantified these levels at 50 DIV (post-slicing) and at 100 DIV using pyrosequencing, comparing the results to the initial mutation load detected in iPSCs (Fig. 1C). While the absolute heteroplasmy levels slightly changed over time in patient organoids, each clone remained within its originally assigned range (i.e., low, medium, or high). In Patient 1, heteroplasmy decreased slightly from 58% to 42% by 100 DIV in one clone, while the high heteroplasmy clone maintained a steady level of 72%. For Patient 2, the low heteroplasmy organoids increased from 15% to 30% by 100 DIV, whereas the medium (from 42% to 49%) and high (from 64% to 70%) heteroplasmy clones showed only a minor increase.

The consistency in heteroplasmy ranges allowed us to effectively assess the impact of the m.3243 A > G mutation load on cell type diversity and homogeneity using single-cell RNA sequencing of 100 DIV organoids. Clustering was performed on non-batch corrected merged samples using Seurat (Fig. 1D), which identified 11 clusters corresponding with major cell types based on known marker genes, apart from one unidentified cluster (Fig. 1D, E).

All identified cellular populations were present in both patient-derived organoids, with similar relative proportions observed across individual samples and patients (Fig. 1D, E). Moreover, immunolabeling analyses showed that the distribution of neurons and the levels of the TUJ neuronal marker were consistent among organoids with varying heteroplasmy (Fig. 1F, G and Supplementary Fig. S1D). Together, these results indicate that heteroplasmy does not significantly affect cellular composition, as both the overall and neuronal cell proportions remain constant within the ranges of mtDNA mutation load.

### Neuronal diversity and maturity are not influenced by heteroplasmy levels

**Fig. 2:**
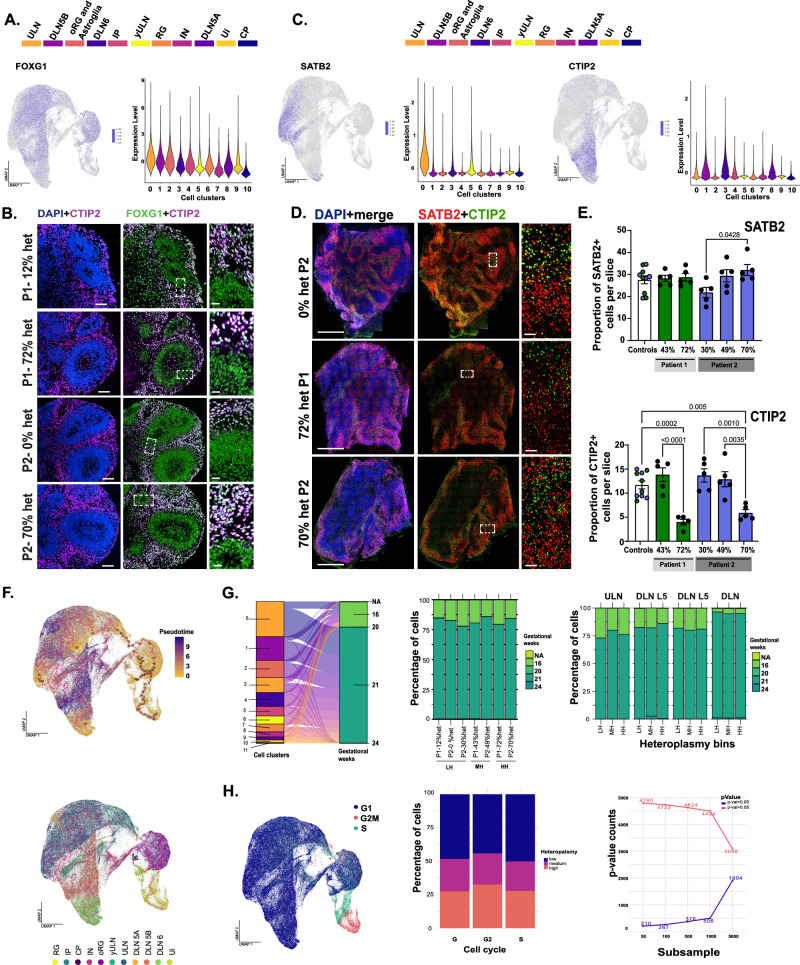
Cortical cell diversity and maturation are unaffected by heteroplasmy and are consistently recapitulated across all heteroplasmic organoids. **A**, **C** Violin plots (left) and UMAP plots (right) illustrating the expression of FOXG1 (forebrain identity), CTIP2 (DLN), and SATB2 (ULN) marker genes by scRNA-seq in DIV 100 organoids. Cluster numbers (x-axis) correspond to colour-coded cell type identities in UMAP plots. **B** Representative immunofluorescence images of cortical plate regions at DIV 75, showing FOXG1 (green) and CTIP2⁺ neurons (violet), with insets highlighting cortical plate organisation. Scale bar = 50 µm. **D** Representative immunofluorescence images of laminar organisation in DIV 100 organoids, with SATB2⁺ (red) marking ULNs and CTIP2⁺ cells (green) marking DLN5 neurons, scale bar = 200 µm. **E** Bar plots quantifying SATB2⁺ and CTIP2⁺ cells, normalised to the total number of cells per ALI-CO cryosection. Data represent the mean ± SEM; *n* = 5 independent ALI-COs per heteroplasmic line and isogenic controls from different batches. Statistical analysis: two-sided one-way ANOVA with Tukey’s post hoc test (overall *p*-values indicated in the graphs). **F** Pseudotime analysis of scRNA-seq data, with branches representing cell-subtype specification (upper) or cluster identities (lower). **G** Projection of transcriptomic data onto a foetal brain reference dataset using scmap. The alluvial plot (left) shows cellular cluster mappings to human gestational ages. Bar plots show proportions of cells assigned to different foetal ages per patient and isogenic control line (middle), and neuronal cell type distributions mapped to foetal developmental stages (right). **H** UMAP of cell cycle gene expression across all 11 cellular populations (left). Bar plot (middle) quantifies cell cycle phase proportions across heteroplasmic groups. Statistical analysis: Pearson’s chi-squared test was used to compare distributions of categorical data. *P*-values have been adjusted using the Benjamini-Hochberg procedure, suggesting no significant differences in cell cycle distribution across heteroplasmic organoids. Source data are provided as a Source Data file.

We investigated whether transcriptomic signatures distinguish distinct cellular subtypes and whether characteristics of these subtypes are affected by heteroplasmy. By 75 days in vitro (DIV), the telencephalic marker FOXG1 showed widespread immunoreactivity in organoids, and by 100 DIV it is broadly expressed across all cellular clusters, confirming forebrain identity (Fig. 2A, B, Supplementary Fig. S2A and Supplementary Data 1). Corresponding with the early developmental stage at 100 DIV, immature cells, such as outer radial glia and astroglial cells (oRG/astroglia, marked by *HOPX*, *TNC, GFAP or AQP4*), inner radial glia (iRG, marked by *TOP2A*) and intermediate progenitors (IP, marked by *EOMES*) have been detected (Supplementary Fig. S2B, C and Supplementary Data 1). Our analysis identified signatures of multiple immature and mature neuronal cell types, including excitatory upper layer neurons (ULN), marked *by CUX2, SATB2*, and *DCX* expression (Fig. 2C, Supplementary Fig. S2B, C and Supplementary Data 1). Deep layer neurons (DLN) with makers for layer 5a, 5b and 6 were also identified based on the expression of *FEZF2/CTIP2*, *CTIP2/PCP4* and *TLE4, FOXP2*, respectively (Fig. 2C and Supplementary Fig. S2B and Supplementary Data 1). In addition, cells with interneuron (IN) identities characterised by *GAD2* and *DLX2* expression were also present (Supplementary Fig. S2B). The cell-subtype-specific transcriptomic signatures suggest that overall cell identities remain largely unchanged, regardless of heteroplasmy levels.

We next evaluated the tissue architecture by immunostaining for upper-layer (SATB2) and deep-layer (CTIP2) neuronal markers (Fig. 2D, E and Supplementary Fig. S2D). Although the overall laminar organisation was preserved in all organoids, evident from the segregation of deep-layer from upper-layer neurons, the proportion of CTIP2-positive cells was significantly reduced in high-heteroplasmy organoids. In contrast, SATB2-positive populations remained unaffected (Fig. 2E). Together, these findings indicate that high m.3243 A > G heteroplasmy preferentially affects deep-layer neuronal populations, while overall neuronal abundance, assessed using the pan-neuronal marker TUJ and the neuronal nuclear marker NeuN, remains largely preserved across heteroplasmy levels (Fig. 1F, G and Supplementary Fig. S2E, F).

Finally, we directly assessed whether the m.3243 A > G heteroplasmy load affects cell type specification, maturation and cell cycle, potentially influencing the readout on m.3243 A > G-driven mechanisms in neuronal pathology. To do so, we analysed the pseudotime trajectories in the control and heteroplasmic organoids using Monocle3^20^. This illustrated very similar transitions from radial glia (RG) towards mature DLN and ULN cell types following the typical developmental sequences in all groups (Fig. 2F and Supplementary Fig. S2G). We then compared the transcriptomic maturity profiles of isogenic controls and heteroplasmic organoids with an age-specific reference dataset deriving from human foetal brain tissue^21^ using scmap^22^ (Fig. 2G and Supplementary Fig. S2H). The maturity profiles across all samples aligned well with foetal brain cell transcriptomic signatures at 21 gestational weeks (140 days), including neurons, which closely matched the in vitro age of organoids (Fig. 2G and Supplementary Fig. S2G). In addition, the cell cycle signatures inferred from merged transcriptomic data revealed that most cells in the various heteroplasmic organoids were uniformly in the G1 phase, characteristic of post-mitotic neurons, while some cells were in the S and G2 phases, corresponding with progenitor cell identities (Fig. 2H). Our findings, therefore, indicate that the m.3243 A > G variant does not cause cell differentiation changes or cell cycle arrest, allowing accurate elucidation of mutation-driven pathologies in mature cortical neuronal populations.

### Cortical organoids with the m.3243 A > G variant display a mitochondrial defect

**Fig. 3:**
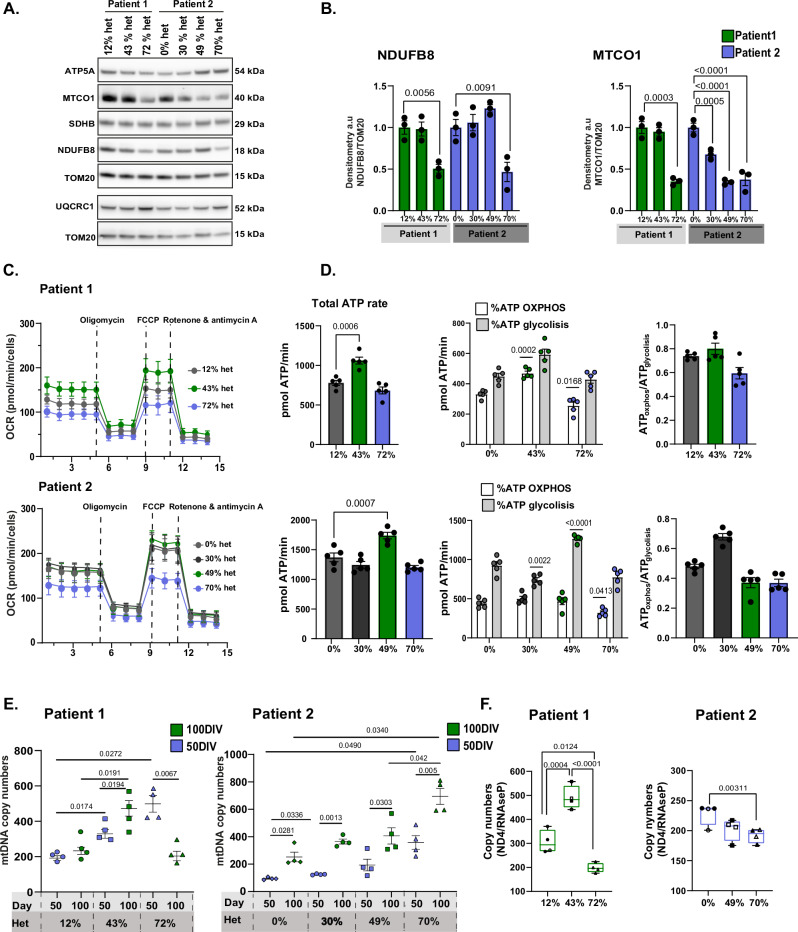
High heteroplasmy m.3243 A > G organoids display a mitochondrial defect. **A** Representative western blot (WB) images for ATP5A, MTCO1, SDHB, NDUFB8, TOM20, and ACTB with molecular weights indicated. **B** Quantification of NDUFB8 (Complex I) and MTCO1 (Complex IV) normalised to TOM20. Data represent the mean ± SEM; *n* = 3 ALI-COs from three independent batches, with two organoids pooled per sample. Statistical analysis: one-way ANOVA with Dunnett’s multiple comparisons test (overall *p*-values indicated in the graphs); **C** Oxygen consumption rate (OCR) in 100 DIV dissociated organoids. The upper panel represents data from Patient 1, and the lower panel data from Patient 2. OCR was measured at basal levels, following 1 µM oligomycin, 1 µM FCCP, and 1 µM rotenone/antimycin A. OCR was normalised to total cell number by Hoechst staining. **D** ATP production rates calculated from Seahorse OCR and ECAR measurements. Bar plots show total ATP production (left), mitochondrial ATP production (ATPOXPHOS; middle), glycolytic ATP production (ATPglycolysis; middle), and the ATP_OXPHOS_/ATP_glycolysis_ ratio (right). The top panels correspond to Patient 1 and the bottom panels to Patient 2. Data represent *n* = 5 dissociated ALI-COs for heteroplasmic and control organoids, with each experiment performed in technical triplicate to confirm trends. Statistical analysis: one-way ANOVA followed by Dunnett’s multiple comparisons (overall *p*-values indicated in the graphs). **E** Dot plot showing individual measurements of mtDNA copy number in whole organoids at 50 and 100 DIV. Data represent the mean ± SEM of *n* = 4 ALI-COs from different batches. Statistical analysis: two-way ANOVA followed by Tukey’s multiple comparisons test (overall *p*-values indicated in the graphs). **F** Box plots showing absolute quantification of mtDNA in neurons sorted from 100 DIV organoids. The centre line indicates the median, box bounds represent the interquartile range (25th-75th percentile), and whiskers extend to minimum/maximum values, with individual data points overlaid. *n* = *2* independent sorting experiments, each from three pooled organoids, with two technical qPCR replicates per sort. Statistical analysis: one-way ANOVA with Tukey’s post hoc test (overall *p*-values indicated in the graphs). Source data are provided as a Source Data file.

To determine how varying heteroplasmy levels influence mitochondrial function in organoids, we first examined OXPHOS protein abundances (Fig. 3A). Organoids with high heteroplasmy exhibited reduced levels of NDUFB8, a Complex I subunit, whereas MTCO1 and MTCO2, Complex IV subunits, were decreased also in medium and high heteroplasmy organoids (Fig. 3A, B and Supplementary Fig. S3B, C). In contrast, SDHB (Complexe II), UQCRC1 (Complex III) and ATP5A (Complex V), remained unchanged across all organoids while MT-ATP6 (Complex V) showed a moderate reduction in high-heteroplasmy organoids (Fig. 3A and Supplementary Fig. S3BC).

These results confirm that the extent of OXPHOS disruption depends on heteroplasmy levels, with Complex I and IV being more susceptible to elevated mutation burden. However, the concordant reduction across mtDNA-encoded proteins may also reflect a broader effect on mtDNA-encoded protein abundance, rather than a strictly complex-specific vulnerability.

Real-time metabolism was monitored by the Seahorse assay, which was performed on cells dissociated from organoid slices. Using the OCR and ECR traces, we calculated ATP production rates as described previously^23,24^. In both patients, total ATP production increased in medium heteroplasmy but remained unchanged in high heteroplasmy organoids at 100 DIV compared to controls. When separating ATP sources, glycolytic ATP production was elevated in medium heteroplasmy organoids, while mitochondrial ATP production was specifically reduced only in high heteroplasmy. As a consequence, the ATP_OXPHOS_/ATP_glycolysis_ ratio decreases with increasing heteroplasmy, indicating a shift in glycolytic reliance (Fig. 3C, D). High heteroplasmy levels were also associated with a tighter coupling of oxidative phosphorylation and a shift toward glycolysis, evident by a decreased proton leak and a lower OCR/ECAR ratio (Supplementary Fig. S3D, E). In contrast, low and medium-heteroplasmy organoids did not exhibit these respiration defects, as the mutation load remains below the pathogenic threshold that can cause mitochondrial dysfunction (Fig. 3C, D and Supplementary Fig. S3C). Because the proportions of mature neurons (NeuN⁺) and progenitor cells (SOX2⁺) were comparable across heteroplasmy levels in organoids at 100 DIV (Supplementary Fig. S2E, F), the OXPHOS and ATP defects observed in high-heteroplasmy organoids are most consistent with intrinsic mitochondrial dysfunction rather than differences in cellular composition.

Next, we examined how cortical organoids regulate mtDNA content in response to metabolic dysfunction at 50 and 100 DIV. At 50 DIV, medium and high heteroplasmy organoids have increased mtDNA copy numbers compared to controls, consistent with a compensatory response to maintain mitochondrial function (Fig. 3E). As organoids mature and accumulate more post-mitotic cells, low heteroplasmy organoids continued to increase mtDNA copy numbers between 50 and 100 DIV, with all medium and P2 high heteroplasmy organoids following a similar pattern (Fig. 3E). In contrast, P1 high heteroplasmy (72%) organoids failed to sustain mtDNA expansion at 100 DIV, suggesting a threshold beyond which further amplification is not possible.

We examined mtDNA copy number regulation specifically in post-mitotic cells by isolating neurons and astrocytes from dissociated 100 DIV organoids using fluorescence-activated cell sorting (FACS) (Supplementary Fig. S3F). In high heteroplasmy neurons, mtDNA copy numbers were significantly reduced compared to controls (40% in P1 and 26% in P2), whereas medium heteroplasmy neurons exhibited more variability, suggesting the presence of compensatory mechanisms (Fig. 3F and Supplementary Fig. S3F). In astrocytes, mtDNA copy numbers decreased both in medium and high heteroplasmy conditions, with a significant reduction (43%) observed only in high heteroplasmy P1 astrocytes (Supplementary Fig. S3G). Importantly, heteroplasmy levels in neurons and astrocytes remain unchanged and are comparable to those observed in the whole organoid (Supplementary Fig. S3H). Together, all these findings highlight a critical heteroplasmy threshold beyond which mitochondrial compensation fails, leading to metabolic dysfunction and cell type-specific vulnerability.

### m.3243 A > G drives transcriptomic signatures of perturbed neuronal homoeostasis

**Fig. 4:**
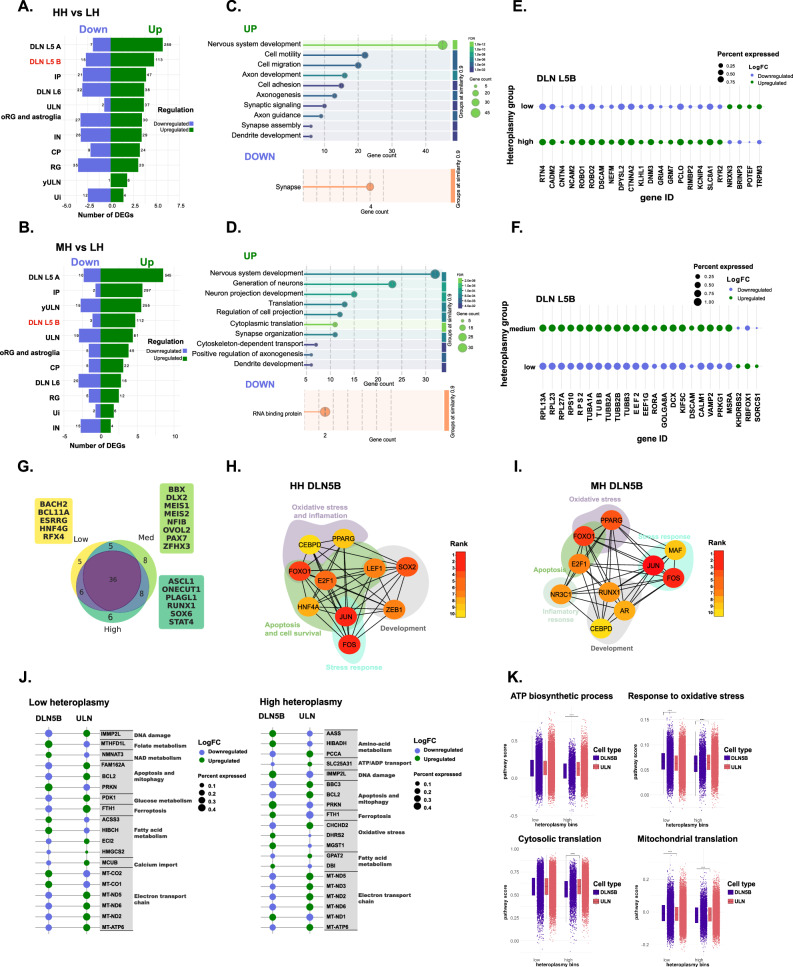
The m.3243 A > G mutation impacts neuronal homoeostasis. **A**,** B** Number of differentially expressed genes (DEGs) per cell type in high heteroplasmy (HH) vs. low heteroplasmy (LH) and medium heteroplasmy (MH) vs. low heteroplasmy (LH) (three slices per organoid line). **C**,** D** Dot plots representing gene set enrichment analysis (GSEA) identifying the top 10 Gene Ontology (GO) terms with the largest term size enriched in DLN5B for both comparisons, performed using STRING^47^. Terms were grouped by similarity, with bubble colour and connecting lines indicating the false discovery rate (FDR) value, while bubble size represents the number of enriched terms per pathway.** E**,** F**. Dot plots showing selected DEGs associated with the enriched GO terms identified in (C, D) for DLN5B in both comparisons. **G** Venn diagram illustrating shared and unique transcription factors (TFs) identified in high, medium, and low heteroplasmy organoid groups. TF analysis was performed using SCENIC on merged data, representing transcription factors across all cell populations. **H**, **I** Core network interaction maps (STRING) of transcription factors with predicted activity, identified using SCENIC. TFs were ranked by Maximal Clique Centrality (MCC) network topology (CytoHubba from Cytoscape), and functional domains were assigned based on gene/protein function (UniProt, https://www.uniprot.org). Node (circle) size corresponds to the MCC score, while colour gradient represents MCC score ranking. **J** Dot plots showing all DEGs mapping to MitoCarta identified in our single-cell RNA sequencing (scRNA-seq) analysis when comparing DLN5B with ULN in both low and high heteroplasmy conditions. Transcripts were categorised into functional domains based on gene/protein function (UniProt, https://www.uniprot.org). **K** Box plots representing GO enrichment scores of major pathways in DLN5B versus ULN for low and high heteroplasmy organoids, derived from *n* = 2 patients per group. The centre line indicates the median, box bounds represent the 25th-75th percentile (IQR), and whiskers extend to 1.5 × IQR. Individual cells are overlaid as data points for visualisation. Statistical analysis: two-sided Wilcoxon rank-sum test comparing pathway score distributions between cell types at the single-cell level (**** *p* < 0.001).

By analysing differentially expressed genes (DEGs) from scRNA-seq data, we examined how mitochondrial dysfunction drives cell type specific changes in organoids. We compared transcriptomic signatures between high (72% het P1, 70% het P2) and low heteroplasmy (12% het P1, 0% het P2, 30% het P2) and between medium (43% het P1, 49% het P2) and low heteroplasmy. In both comparisons, intermediate progenitors consistently ranked among the top three populations with the highest number of DEGs. A striking difference emerged amongst neuronal populations. Deep-layer neurons (DLN5A and DLN5B) were most affected in high heteroplasmy, while DLN5A cells and developing upper-layer neurons (ULN) were the predominantly involved population in medium heteroplasmy (Fig. 4A, B, Supplementary Data 2 and 3).

Gene Ontology (GO) analysis revealed that high heteroplasmic DLN5B neurons show significant alterations in pathways related to cell adhesion, axon/dendritic development (*RTN4, CADM2, CNTN4, NCAM2, ROBO1/2, DSCAM*), and cytoskeletal remodelling (N*EFM, DPYSL2, CTNNA2, KLHL1, DNM3*), suggesting axonal disruption and degeneration. The upregulation in gene expression of glutamate receptor subunits (*GRIA4, GRM7*), presynaptic scaffolding proteins (*PCLO, RIMBP2*), and expression changes in ion channel (*KCNH7, KCNIP4*) and calcium exchanger genes (*SLC8A1, RYR2*) further indicate synaptic dysfunction and altered neuronal excitability (Fig. 4C, E). In contrast, in medium heteroplasmy DLN5B neurons exhibited a stress-adaptive response, characterised by an enrichment of cytosolic translation related transcripts, including ribosomal proteins (*RPL13A, RPL23, RPL27A, RPS10, RPS2*) and elongation factors (*EEF2, EEF1G*). We observed an increased expression of tubulin genes (*TUBA1A, TUBB, TUBB2A, TUBB2B, TUBB3*), suggesting active cytoskeletal reorganisation that supports axonal and dendritic development (Fig. 4D, F).

Transcription factor (TF) inference analysis^25^ across the merged data identified 36 common TFs linked to organoid development and maturation (Supplementary Fig. S4A). In high heteroplasmy, we identified six uniquely expressed TFs, including *PLAGL1* and *STAT4*, which are associated with apoptosis and inflammation, supporting a pathogenic environment. Medium heteroplasmic cells express unique TFs predominantly involved in developmental pathways and cell maintenance, implying engagement of adaptive mechanisms to preserve neuronal function and regional identity (Fig. 4G). Further TF activity analysis in high and medium heteroplasmic DLN5B cells using STRING networks and CytoHubba Maximal Clique Centrality analysis (embedded in Cytoscape)^26^ revealed a core set of stress-responsive TFs (*PPARG, FOXO1, E2F1, JUN, FOS*). High heteroplasmic neurons uniquely express additional TFs (*LEF1, HNF4A, ZEB1*) that reinforce apoptotic and oxidative stress signalling, whereas medium heteroplasmy neurons engage factors such as *NR3C1* and *PPARG* that can promote stress resilience (Fig. 4H, I and Supplementary Fig. S4B).

To explore the potential causes for the cell stress changes predicted predominantly for DLN5B neurons, we compared their transcriptomic profiles with those of ULN neurons in control and high heteroplasmy cells (Supplementary Data 4 and 5). Control DLN5B neurons displayed a more oxidative metabolic profile with increased Complex IV transcripts (*MT-CO1, MT-CO2*), lower Complex I transcripts (*MT-ND2, MT-ND4, MT-ND6*) and enriched fatty acid metabolism, compared to ULN neurons, which are enriched for glycolysis-related GO terms (Fig. 4J, K and Supplementary Fig. S4C, D). High heteroplasmy DLN5B neurons displayed metabolic disruptions compared to ULN, with a significant downregulation of ATP biosynthesis pathways together with decreased response to oxidative stress and an increase in ferroptosis (*FTH1*), and DNA damage markers (*IMMP2L*), suggesting increased cellular stress and increased apoptosis. Additionally, mitochondrial and cytosolic translation-related GO terms were significantly overrepresented in high heteroplasmy DLN5B neurons compared with ULN, indicating cell type-specific translational dysfunction (Fig. 4K). Together, these findings demonstrate that DLN5B neurons are more vulnerable to mitochondrial dysfunction, oxidative stress, and apoptotic signalling under high heteroplasmy compared to both low heteroplasmy conditions and high heteroplasmy ULN neurons, aligning with their predominant loss in patient-derived organoids.

### m.3243 A > G high heteroplasmy promotes apoptosis of deep layer neurons

**Fig. 5:**
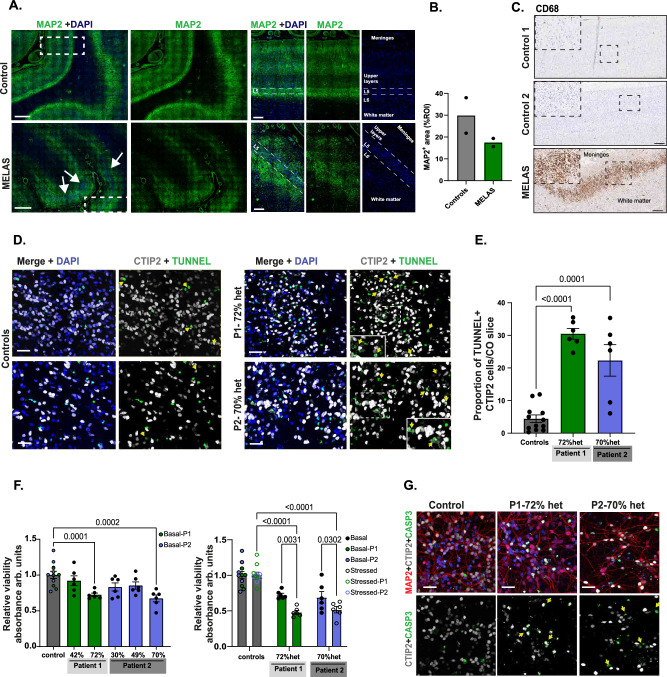
High heteroplasmy m.3243 A > G mutation leads to neuronal apoptosis in deep layer neurons. **A** Representative immunofluorescence image of human brain tissue from one m.3243 A > G individual and age-matched controls, stained for MAP2 (microtubule-associated protein 2, green) and DAPI (blue). Dotted white lines in the right panels marking layer 5 neurons. *n* = *1*. Scale bar = 200 µm (right) and 50 µm (left). **B** Bar plot showing MAP2-positive area as a percentage of total tissue area in control and MELAS post-mortem cortex. Two ROIs were analysed per sample. Data shown as individual ROI values with bars representing the mean. **C** Representative immunohistochemistry images of cortical tissue from one m.3243 A > G individual and two age-matched controls stained for CD68 (activated microglia). Insets highlight CD68-positive regions. Scale bar = 200 µm, **D** Representative immunofluorescence images of 100 DIV cortical organoids with high heteroplasmy and isogenic controls, showing CTIP2⁺ cells (grey) and TUNEL⁺ nuclei. Scale bar = 100 µm. **E** Bar plots quantifying TUNEL⁺ cells co-localising with CTIP2. Data represent the mean ± SEM; *n* = *6* independent ALI-COs per heteroplasmic line and isogenic controls. Statistical analysis: one-way ANOVA with Tukey’s post hoc test, with overall *p*-values indicated in the graphs. **F** Left-relative cell viability by PrestoBlue assay in 2D deep-layer neurons at DIV 30 under basal conditions. Data represent the mean ± SEM, *n* = *6* from three independent batches of neurons. Statistical analysis: one-way ANOVA with Dunnett’s post hoc test, with overall *p*-values indicated in the graphs; Right- relative cell viability under basal and stressed conditions following acute stimulation with 10 µM GSK-2837808A + 0.1% AlbuMAX for 12 h. Data represent the mean ± SEM; *n* = *6* from three independent batches of neurons. Statistical analysis: two-way ANOVA with Tukey’s multiple comparisons test, with overall *p*-values indicated in the graphs, **G** Representative immunofluorescence images of CTIP2^+^ cells (grey), cleaved CASP3 (green) and MAP2 (red) in isogenic control and high heteroplasmy glutamatergic 2D neurons. *n* = *3* biological replicates. Scale bar = 50 µm. Source data are provided as a Source Data file.

To extend our organoid findings to human pathology, we examined postmortem brain tissue from a 70-year-old male MELAS patient carrying the m.3243 A > G variant with 27% heteroplasmy detected in blood and 82% in the brain tissue. The patient initially presented with mitochondrial myopathy, ophthalmoparesis, and fatigable weakness, with muscle biopsy showing abnormal myofibers, COX-/SDH-positive fibres, and selective type II fibre atrophy (Supplementary Fig. S5A). Following the development of acute neurological symptoms and stroke like episodes (SLEs), the patient died within 4 months. Brain autopsy revealed severe neuronal loss in the occipital lobe and associated glial fibrillary acidic protein (GFAP) immunoreactivity, suggesting a marked glial cell response, compared with two age-matched control brain tissues deriving from a 57-and an 85-year-old male patient) (Supplementary Fig. S5B). Specifically, layer V, identified by its dense DAPI staining, showed a marked reduction in MAP2 immunoreactivity and the lack of cells with deep pyramidal neuronal morphology (Fig. 5A, white arrows) when compared to the control cortical samples. The upper layer also showed some decrease in MAP2^+^ neurons, while layer VI appeared relatively preserved. Consistent with this, MAP2 positive areas were reduced in MELAS cortex compared to controls (22–38% in controls versus 15–19% in MELAS, Fig. 5B). In addition, the presence of CD68 immunoreactivity revealed increased microglial activation in deep cortical layers of the MELAS tissue (Fig. 5C). Although this observation is based on a single post-mortem sample, it is broadly consistent with the selective vulnerability of deep layer neurons observed in MELAS organoids (Fig. 2D, E), suggesting a possible shared mitochondrial mechanism.

We next determined whether the neuronal loss is a result of apoptotic cell death in MELAS organoids. Apoptosis was assessed in organoids with high heteroplasmy and isogenic controls using a terminal deoxynucleotidyl transferase dUTP nick end labelling (TUNEL) assay (Fig. 5B). We found an increased number of TUNEL-positive cells colocalising with CTIP2 immunoreactivity, indicating that apoptosis contributes to the reduced DLN5 population in high heteroplasmic organoids (Fig. 5D, E). In contrast, quantification of SATB2⁺/TUNEL⁺ proportion of cells shows no change in high heteroplasmy organoids (Supplementary Fig. S5C and D). Consistent with intact generation of cortical projection neuron subtypes, CTIP2 and SATB2 immunostaining at 70 DIV showed comparable proportions of DLN and ULN neurons in control and high heteroplasmy organoids (Supplementary Fig. S5E and F). Thus, our data support a selective late-stage loss of CTIP2⁺ DLN neurons under high m.3243 A > G heteroplasmy.

To further explore the contribution of mtDNA heteroplasmy-related cell death in this specific neuronal population, we generated CTIP2^+^ DLN5 neuronal mono-cultures obtained from a homogeneous pool of neuronal progenitors (Supplementary Fig. S5G–I). By day 30, high heteroplasmy neurons showed decreased viability compared with medium and low heteroplasmy cells (Fig. 5F). The increased immunoreactivity of cleaved CASPASE3 in CTIP2 neurons supported their apoptotic cell death (Fig. 5G). LDHA blockade evoked metabolic stress, and lipid supplementation driven induction of mitochondrial function revealed further decrease in viability in high heteroplasmy neurons, while medium and low heteroplasmy cells remained mostly unaffected (Fig. 5F and Supplementary Fig. S5J). These findings corroborate that the death of high heteroplasmy DLN5B neurons is exacerbated by mitochondrial stress.

### High heteroplasmy alters mitochondrial morphology and axon integrity in deep layer neurons

**Fig. 6:**
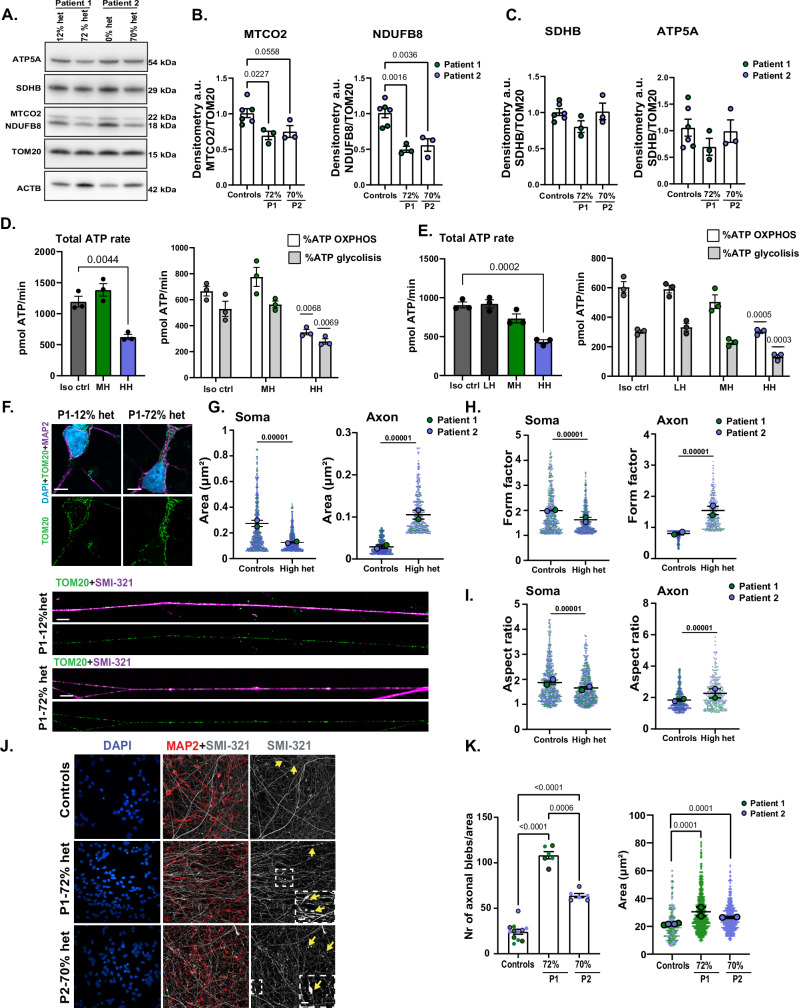
Deep layer neurons display mitochondrial defect. **A** Representative WB images for ATP5A (Complex V), SDHB (Complex II), MTCO2 (Complex IV), NDUFB8 (Complex I), TOM20, and ACTB in 30 DIV glutamatergic neurons, with molecular weights indicated. **B**,** C** Quantification of MTCO2, NDUFB8, SDHB, and ATP5A, normalised to TOM20 and ACTB, was used as a loading control. Data represent the mean ± SEM; *n* = *3* from independent batches. Statistical analysis: one-way ANOVA with Dunnett’s post hoc test, with overall *p*-values indicated in the graphs. **D**,** E** ATP production rates from Seahorse OCR and ECAR measurements in DLN neurons (LH, low heteroplasmy; MH, medium heteroplasmy; HH, high heteroplasmy). Bar plots show total, mitochondrial (ATP_OXPHOS_) and glycolytic (ATP_glycolysis_) ATP production and the ATP_OXPHOS_/ATP_glycolysis_ ratio. Left panels, Patient 1; right panels, Patient 2. Data represent the mean ± SEM; *n* = *3*, from three independent batches. Statistical analysis: one-way ANOVA with Dunnett’s post hoc test (overall *p*-values indicated in the graphs). **F** Representative images showing MAP2 (magenta) and TOM20 (green) in 2D control and high-heteroplasmy neurons. Scale bar = 50 µm; Images of axonal mitochondria (TOM20 green) and axonal neurofilament marker SMI-321 (magenta) in 2D neurons from controls and high-heteroplasmy lines. Scale bar = 50 µm. **G**–**I** Dot plots of mitochondrial morphology in soma and axons, assessing mitochondrial area, form factor, and aspect ratio. Small dots represent individual mitochondria; large dots represent the median per cell line, with error bars indicating the range between cell lines per group. *n* = *4* cell lines (two control, two high-heteroplasmy) from two independent batches. Statistical analysis: unpaired two-tailed Mann-Whitney test. **J** Immunofluorescence images of axons (SMI-321, grey) and dendrites (MAP2, red) in control and high-heteroplasmy neurons, with insets highlighting axonal fragmentation and blebs. Scale bar = 50 µm. **K** Bar plots quantifying axonal bleb number per area (left) and bleb area (right). Data represent the mean ± SEM; *n* = *4* (two control, two high-heteroplasmy lines, two batches). Statistical analysis: one-way ANOVA with Tukey’s test (left); Kruskal-Wallis with Dunn’s test (right). Overall *p*-values indicated in the graphs. Source data are provided as a Source Data file.

To explore further mitochondrial abnormalities in deep-layer neurons, we compared OXPHOS protein levels and mitochondrial morphology in cortical neuronal mono-cultures with high heteroplasmy versus isogenic control cells. Western blotting revealed a consistent reduction in MTCO1 (Complex IV) and NDUFB8 (Complex I) in high heteroplasmy deep-layer neurons, while SDHB (Complex II) and ATP5A (Complex V) remained largely unchanged compared to controls (Fig. 6A–C). These results align with our findings in cortical organoids (Figs. 3A, B and 6A, C), supporting the mitochondrial defects in high heteroplasmy neurons. Seahorse analysis demonstrated a heteroplasmy-dependent reduction of total ATP production. (Fig. 6D, E, Supplementary Fig. S6 A-D). Although OXPHOS remains the primary source of ATP, its contribution was significantly decrease in high heteroplasmy neurons. The absence of a compensatory increase in glycolytic ATP indicates a failure of metabolic reprogramming and highlights an intrinsic vulnerability of these neurons to mitochondrial dysfunction (Fig. 6D, E).

To determine whether these molecular alterations in OXPHOS and bioenergetics were accompanied by structural changes, we examined mitochondrial morphology in DLN using TOM20 as a mitochondrial marker, MAP2 for dendrites (cell body), and SMI-32 for axons (Fig. 6F–I and Supplementary Fig. S6E). In the soma, mitochondria in high heteroplasmic neurons displayed reduced area, indicative of smaller, fragmented mitochondria (Fig. 6F–G). This was accompanied by changes in form factor and aspect ratio, further supporting abnormal mitochondrial morphology (Fig. 6H–I). In contrast, axonal mitochondria in high heteroplasmy neurons displayed increased area, particularly accumulating in axonal blebs, alongside elevated form factor and aspect ratio (Fig. 6F–I), suggesting a more elongated mitochondrial morphology.

The abnormal mitochondrial morphology and axonal distribution, along with scRNA-seq data indicating axonal dysfunction, in high heteroplasmy deep-layer neurons (Fig. 4C), prompted us to examine axonal structure. Immunofluorescence of SMI-32, an axonal label, revealed a significantly increased number of axonal blebs, with enlarged bleb area compared to controls (Fig. 6J, K). Our findings indicate that mitochondrial dysfunction in high heteroplasmy neurons contributes to axonal structural abnormalities with signatures of early degeneration, which highlights a mitochondrial stress-induced axonal vulnerability in deep-layer neurons.

## Discussion

Here, we developed a 3D human cortical brain organoid model carrying the heteroplasmic m.3243 A > G mtDNA mutation, the most common pathogenic mtDNA variant^27^. So far, one recent study used a human 3D brain tissue model harbouring a nuclear mutation in *SURF1*^28^ to study mitochondrial dysfunction, while cortical organoid platforms have not been utilised to explore mtDNA mutations^16^. The use of these advanced human systems is a pre-requisite for studying common mtDNA mutations due to the lack of animal models. Importantly, organoid slice culture preparations allow long-term cultures, cell type diversity and maturation, improving their reliability to model human diseases^17,19,29^.

Our 3D human cortical brain organoid slice model of m.3243 A > G successfully recapitulated mature neuronal phenotypes while exhibiting distinct molecular pathological characteristics of mitochondrial dysfunction. In this study, we uncovered key molecular mechanisms underlying MELAS, the most severe phenotype of m.3243 A > G observed in patients, and identified other brain related neuronal pathologies associated with this common mtDNA mutation. Using isogenic and mutant cortical organoids derived from seven iPSC clones from two patients with low, medium, and high heteroplasmy levels, we systematically examined how different mutation burdens affect neuronal homogeneity, maturity, and diversity, as well as their impact on neuronal cell homoeostasis.

Heteroplasmy levels in our organoids remained stable throughout their formation, showing consistency across different organoids and batches, suggesting no major selective pressure for or against the mutation. Likewise, neuron- and astrocyte-specific mutation loads were comparable, suggesting that heteroplasmy remains uniform across different mature cell populations within the organoids. This stability aligns with previous studies indicating that rapid shifts in heteroplasmy primarily occur during the reprogramming of fibroblasts into iPSCs, whereas heteroplasmy levels remain relatively stable throughout neuronal development^12,13^. In human post-mortem studies, brain heteroplasmy levels of the m.3243 A > G mutation have been reported to be high in both symptomatic and asymptomatic individuals, suggesting that while heteroplasmy contributes to the clinical manifestations of MELAS, it is not the sole determinant of disease severity^30,31^. Additional factors such as mtDNA copy number, nuclear genetic background, and environmental influences likely play critical roles in modulating the phenotype^9^. These findings highlight the complexity of mitochondrial disease and suggest that stable heteroplasmy in organoid models provides a useful system for studying cell type-specific mitochondrial dysfunction without the confounding effects of dynamic heteroplasmy shifts.

Our findings demonstrate that high heteroplasmy levels do not affect cellular composition or heterogeneity, as all 11 identified neuronal populations with single-cell RNA sequencing were present in both control and m.3243 A > G organoids. These organoids also contain mature neuronal subtypes, including distinct deep- and upper-layer cortical architecture. Furthermore, no evidence of cell cycle arrest was observed in high heteroplasmy organoids compared to medium or low heteroplasmy. While mitochondrial dysfunction can influence neuronal maturation^32,33^, our results suggest that these effects may be more subtle in m.3243 A > G disease or only become apparent at higher heteroplasmy thresholds ( < 80% het). This aligns with the milder and later onset manifestation of MELAS in adults^9,34^, in contrast to severe early childhood-onset mitochondrial diseases such as Leigh Syndrome due to *SURF1* mutations, where brain organoids showed that morphogenesis is profoundly disrupted^28^.

Mitochondrial dysfunction correlated with heteroplasmy load in cortical organoids, with high-heteroplasmic organoids showing the most severe OXPHOS defects. Complex I expression was consistently reduced, while complex IV levels varied across heteroplasmy loads, mirroring findings from m.3243 A > G patient brain autopsies^31^. Biochemical assays confirmed metabolic impairment in high-heteroplasmy organoids with limited ability to adapt to metabolic stress. This vulnerability was further supported by studies on 2D neurons, where increased mitochondrial activity and stress led to decreased viability, likely due to apoptosis from excessive metabolic burden. In contrast, medium heteroplasmy organoids with normal mitochondrial function and one high heteroplasmy organoid (P2-70%) exhibited increased mtDNA copy numbers, suggesting a compensatory mechanism to sustain OXPHOS by preserving wild-type mtDNA. This effect appears limited beyond a certain threshold, leading to irreversible mitochondrial defects. Despite similar heteroplasmy levels in high heteroplasmy P1 and P2 at 100 DIV, their distinct mtDNA trajectories suggest that heteroplasmy levels have a strong influence on mtDNA expansion at earlier stages. A similar adaptation has been observed in dopaminergic neurons, where increased mtDNA copy number have been proposed as a compensatory response to age-related mitochondrial dysfunction^35^. Importantly, since heteroplasmy levels are similar between neurons, astrocytes, and whole organoids, copy number alterations are unlikely to be the main factor driving the mtDNA differences between different cell types and organoid preparations. This suggests that mtDNA maintenance is regulated in a cell type-specific manner, with distinct mechanisms operating in post-mitotic and proliferative cells.

Another important finding of our study was the predominant loss of deep-layer neurons (DLNs) in high-heteroplasmy m.3243 A > G organoids, while upper-layer neurons (ULNs) remained largely preserved. Our scRNA-seq analysis suggests that this vulnerability is linked to intrinsic transcriptional differences between these main neuronal cell types that make DLNs more prone to apoptosis, oxidative stress, and metabolic dysfunction. Specifically, DLNs exhibited dysregulation in pathways associated with cell adhesion, cytoskeletal remodelling, axon and dendritic homoeostasis when compared to isogenic control neurons. These transcriptional changes were reflected in our functional assays, which showed increased apoptosis and axonal degeneration in DLNs, alongside alterations in axonal mitochondrial morphology. The increased susceptibility of DLNs to mitochondrial dysfunction can be attributed to their distinct anatomical and physiological properties. Amongst cortical projection neurons, *CTIP2*-expressing DLN5 neurons extend long-range connections to the spinal cord, brainstem, and thalamic regions^36,37^. Maintaining such extensive networks requires high metabolic energy, partly due to their longer axons and depolarised resting membrane potentials. This increased bioenergetic demand likely makes DLNs particularly vulnerable to disruptions in ATP production and oxidative phosphorylation caused by the m.3243 A > G mutation. Importantly, DLNs are also affected in other neurological disorders such as amyotrophic lateral sclerosis, Parkinson’s disease, Alzheimer’s, epilepsy, further supporting their inherent susceptibility to disease and pathological changes^17,38–41^.

In comparison, ULNs have shorter axons and primarily project local^42^, they were preserved in high heteroplasmic organoids, suggesting a greater resilience to mitochondrial dysfunction. Direct comparison between high heteroplasmic DLN5B and high heteroplasmic ULNs revealed that DLN5 neurons exhibited greater metabolic disruption, including downregulation of ATP biosynthesis pathways and mitochondrial/cytosolic translation-related transcripts. In addition, DLN5B neurons showed elevated expression of oxidative stress markers, ferroptosis-associated genes, and DNA damage markers, indicating higher levels of mitochondrial stress and reduced cellular resilience compared to ULNs. These findings suggest that ULNs are more capable of withstanding mitochondrial dysfunction, potentially due to a more effective metabolic compensation mechanism or lower energy requirements. Together, our results highlight a crucial link between neuronal subtype-specific metabolic demands and their differential vulnerability to mitochondrial dysfunction.

To correlate our findings with brain pathology, we examined autopsy data from a patient who died of m.3243 A > G-related SLEs and detected severe neuronal loss. Postmortem analyses of m.3243 A > G patients have consistently revealed cortical lesions and laminar necrosis (often affecting layer III and V) in parietal, lateral temporal, and occipital cortices, leading to clinical manifestations such as cortical blindness and homonymous hemianopia^31^. These findings suggest a selective vulnerability of specific neuronal populations, mirroring the deep-layer neuronal loss observed in our organoid model. Patients with high heteroplasmic m.3243 A > G often experience SLEs with fluctuating metabolic lesions in cortical-subcortical areas, commonly associated with complex epileptic activity and progressive brain atrophy^10^. Although advanced neuroimaging techniques such as 7 Tesla MRI and PET with mitochondrial ligands have been used to study MELAS patient brains^43,44^, these approaches lack the resolution to identify cell type-specific neuronal dysfunction. This underscores the need for organoid models, which provide a unique platform for dissecting the dynamic changes of mitochondrial pathology at the cellular level and can facilitate therapeutic development targeting specific neuronal populations.

A limitation of our study is that we primarily focused on neurons and not on non-neuronal cell types, such as glial cells, that may also contribute to the phenotype observed in m.3243 A > G patients. To fully understand their role, future studies should investigate glial dysfunction using older organoids ( > 150 DIV), which allow for more advanced glial maturation. In addition, since m.3243 A > G patients frequently present with seizures, incorporating more complex organoid models that include GABAergic interneurons will be essential for studying electrophysiological dysfunction. Such models, combined with multi-electrode array (MEA) recordings, will provide deeper insights into the mitochondrial aspects of epilepsy and neurodegeneration. Future studies integrating single-cell resolution approaches should help refine our understanding of neuronal and glial susceptibility to mitochondrial dysfunction.

Overall, our study provides a mechanistic insight into cell type-specific mitochondrial defects caused by m.3243 A > G in the brain, revealing how neuronal vulnerability is shaped by metabolic stress and bioenergetic demands. By leveraging human brain organoids, we established a powerful platform for testing potential treatments, including emerging mtDNA editing technologies^6^. Beyond therapeutic development, understanding mitochondrial dysfunction at the cellular level is critical not only for mitochondrial diseases but also for neurodegenerative, neurodevelopmental and psychiatric disorders, where mitochondrial deficits contribute to disease progression. We anticipate that using these human platforms will help to understand how mitochondrial function affects neuronal resilience, and degeneration, which could open new avenues for targeted interventions.

## Methods

### Ethical approval

Human brain autopsy of a patient with MELAS syndrome and age and sex match controls were studied. All patients have been consented for using their clinical data and biological samples for this research as part of the “Genotype and phenotype in inherited neurodegenerative diseases” study (REC ID: 13/YH/0310, Yorkshire & Humber – Bradford Leeds Research Ethics Committee).

The biological samples have been securely stored in approved research facilities at the University of Cambridge or within the Neuropathology Laboratory of the Cambridge University Hospitals NHS Foundation Trust.

### iPSC culture

Human induced pluripotent stem cells (iPSC) were a generous gift from Valeria Chichagova, David Steel and Majlinda Lako, Newcastle University, Newcastle, UK (Patient 1 clones 12, 58 and 72%)^45^ and Tamas Kozicz and Eva Morava-Kozicz from Icahn School of Medicine at Mount Sinai, New York, US (Patient 2 clones 0, 15, 42 and 64%)^12^ with additional details in Table S1. Both iPSC lines were derived from female patients, as confirmed by clinical records. Given the limited sample size (*n* = 2), sex-disaggregated analysis was not performed. All cell lines were adapted and cultured in mTeSR (Stemcell Technologies, 100-0276) on plates coated with Geltrex (Thermo Fisher Scientific, A1413302). Media was changed daily, and colonies were passaged using ReLeSR (Stemcell Technologies, 100-0483) according to the manufacturer’s protocol when confluency reached 60–70%. For cryopreservation, cells were frozen in 90% mTeSR Plus containing 10% dimethyl sulfoxide-DMSO (Sigma Aldrich, D2650).

### Generation of cerebral organoids and air liquid interface slices (ALI-COs)

For the generation of cortical organoids, we have employed a method previously published^17,19^ utilising the STEMdiff Cerebral Organoid kit (Stemcell Technologies, 8570). Briefly, iPSCs were detached using Accutase at 37 °C for 5 min and then washed using mTeSR and 18,000 cells were plated in bottom rounded dishes in Media 1 + Supplement A. Media change was performed at day 3. At day 5 embryoid bodies (EBs) were moved in Media 1 + Supplement B, which supports the formation of neuroectoderm. At day 7 EBs were embedded in 20 µL of Matrigel and put in Media2+Supplement C and D until day 10. From day 10 onwards, organoids were moved to Media 2 + Supplement E and organoids kept on an orbital shaker. Media was changed every 4 days, and from 35 DIV onwards, Matrigel (Corning, 354277) was added in 1:50 dilution to achieve a polarised cortical plate formation. Between 45–50 DIV 300 µm ALI-COs were prepared using a vibratome and placed on Millicell-Cell culture inserts Merck Millipore, PICM0RG50), 30 mm, inserts. After slicing, ALI-COs were fed daily using a Slice Media containing Neurobasal medium (Thermo Fisher Scientific, 21103049) supplemented with 1 × B27 (Thermo Fisher Scientific, 17504044) supplement, 0.45% (w/v) glucose (Sigma-Aldrich, G8769), 1 × Glutamax (ThermoFisher Scientific, 35050038) and 1% antibiotic–antimycotic (ThermoFisher Scientific, 15240062).

### Organoid dissociation into single cells

Organoid cell dissociation was performed using both mechanical and enzymatic methods, similar to previously described^5^. Organoids were transferred into a dish containing 1xdPBS (Sigma Aldrich, D8537). Washed slices were transferred into a gentleMACS C tube (Miltenyi, 130-093-237) containing 2 ml of papain solution (20 units per ml; Worthington, PAP2) and ran on a gentleMACS Octo dissociator (Miltenyi) using the default ABDK programme. After dissociation, the cell suspension was diluted with dPBS containing 0.5 mg ml^−1^ DNAse (Sigma Aldrich, 11284932001) and then spun down at 300 × *g* for 5 min. The cell pellet was resuspended and filtered using a 70 μm strainer (Miltenyi, 130-098-462) and centrifuged again. Resulted cell suspension was centrifuged using the above described conditions and then resuspended in the appropriate buffer. For scRNA seq, the cell pellet was resuspended in dPBS (445 cells per µl) containing 0.04% BSA (Sigma, A9418). For the Seahorse experiment, cells were resuspended in N2B27 media containing 10 μm Y-27632 (Tocris, 1254/10) and plated on Seahorse plates (100,000 cells/well) pre-coated with polyethylenimine-PEI (Sigma Aldrich, P3143) and Geltrex.

### FACS sorting of neurons and astrocytes from organoids

Cell sorting was performed as previously described^46^. Briefly, organoids were washed in 1x dPBS (Sigma Aldrich, D8537), before being placed into gentleMACSTM C tubes (Miltenyi, 130-093-237) in 2 ml of TrypLE (Thermo Fisher Scientific, 12605010) for tissue dissociation. Cells were stained and sorted as described previously^46^. Briefly, cells were sorted using a BD FACSAria™ Flow Cytometer. Automatic normalisation was applied using individually stained samples and a negative control. The gating was determined using an isotype control (mouse IgG1 antibody). Cells were filtered for doublets and debris, and only HEPACAM positive astroglia or L1CAM positive neurons were retained. Post-sorting, samples were centrifuged at 2000 x *g* for 1 min at 4 °C, followed by flash freezing on dry ice and subsequent storage at − 80 °C for long-term preservation.

### Seahorse assay

We measured the oxygen consumption rate (OCR) using the Seahorse XFe96 Analyser following the manufacturer’s instructions. OCR was measured at basal levels, followed by supplementation with 1 µM oligomycin, 1 µM FCCP, and 1 µM rotenone/antimycin A. Three slices from different organoids were dissociated as described above, and then 100,000 cells/ well were seeded in a Seahorse 96-well plate (Agilent Technologies) (minimum 20 wells per group). Cells were left for 3-5 days to recover after dissociation in N2B27 media. Prior to measuring cultures were switched to Seahorse XF DMEM Medium pH 7.4, supplemented with 5 mM Glucose, 1 mM sodium pyruvate, 2 mM L-Glutamine. Cells were maintained at 37 C in a CO_2_-free incubator for one hour prior to OCR measurement. We measured simultaneously mitochondrial respiration (oxygen consumption rate, OCR) and anaerobic glycolysis (extracellular acidification rate, ECAR) using the sequential introduction of oligomycin (Sigma-Aldrich, St. Louis, MO, USA, #C2920-10MG), and then rotenone (Sigma-Aldrich, St. Louis, MO, #83-794) and antimycin A (Sigma-Aldrich, St. Louis, MO, USA, #1397-94-0) (all products at 1 µM). The addition of these drugs allowed us to calculate: basal OCR level, basal ECAR level, maximal respiration, and ATP production, as described in detail before. Data was normalised to total DNA (Hoechst 33342, 2 μM) and analysed using WAVE software (Agilent Technologies).

ATP production rates from oxidative phosphorylation and glycolysis were estimated from Seahorse XF measurements using an integrated proton efflux-based framework. ECAR values were first converted to proton efflux rates using the Agilent buffer factor for XF DMEM assay medium. Glycolytic acidification was then calculated based on the established lactate:H⁺ stoichiometry. Respiratory acidification was corrected using the CO₂-derived H⁺:O₂ relationship, and mitochondrial ATP production was calculated using a P/O₂ ratio of 5.45, as described by Mookerjee et al. and Desousa et al^23,24^. This approach enables quantitative separation of ATP generated by oxidative phosphorylation and glycolysis from standard Seahorse measurements.

### Generation of 2D cortical neurons

Neuronal induction was performed based on a protocol described by Maroof et al.^6^ When iPSC have reached 70% confluency cells were detached using Accutase as described above and then plated in a 6 well pate pre-coated with Geltrex. Cells were kept in mTeSR supplemented with 10 μm Y-27632 for 24 h after which the media was switched with neural induction media NI containing N2B27 media supplemented with 2 µM XAV939 (Tocris 3748), 100 nm LDN193189 (Sigma-Aldrich SML0559) and 10 µM SB431542 (Tocris 1614). Daily media changes were performed, and after 10 days were detached using Accutase (as described above) and replated on a T25 flask coated with Geltrex in N2B27 media. After 5 days NPCs were replated (using Accutase) in 6 well plates Geltrex pre-coated plates and maintained in N2B27 supplemented with 10 nm FGF2 (Peprotech 100-18B).

For neuronal differentiation, NPCs were plated at 50,000 cells/cm^2^ on PEI and Geltrex pre-coated plates in N2B27 media supplemented with 10μm Y-27632. 24 hours after plating cell media was replaced with N2B27 containing 10 µm Compound E which induced cell differentiation. Cell media was replaced every 3 days, and cells were kept in Compound E for 7 days. Afterwards, cells were slowly transitioned from N2B27 to BrainPhys (StemCell Technologies, 05790) supplemented with 1X SM1 without vitamin A (StemCell Technologies 100-1282), 1X N-2 Supplement (Thermo Fisher 17502048), and 10 ng/ml BDNF (PeproTech 450-02). Cells were matured for a total of 30 days.

### Cell viability

Cell viability was measured using the PrestoBlue assay (Thermo Fisher Scientific, A13261), following the manufacturer’s recommendation. Fluorescence readings were obtained using the BMG FLUOstar Omega Microplate Reader (BMG Labtech) according to the manufacturer’s specifications. Blank correction was performed using BrainPhys medium containing 5% PrestoBlue incubated without cells, and values were recorded for further analysis.

For metabolic stress conditions, the same cells were treated with 10 µM GSK-2837808A (Tocris, 5189) and 0.1% AlbuMAX (Thermo Fisher Scientific, 11021029) in BrainPhys medium for 12 h before performing the PrestoBlue assay. Fluorescence measurements were taken as described for the basal conditions.

### m.3243 A > G mtDNA heteroplasmy measurements

DNA was extracted from whole organoid slices using the DNeasy Blood and Tissue Kit from QIAGEN (69504) according to the manufacturer’s protocol and quantified using a NanoDrop spectrophotometer (Thermos Scientific). Heteroplasmy measurements were performed using the QIAGEN Q48 Autoprep workstation system. A section of the mitochondrial genome containing the mutation of interest (m.3243 A > G) was amplified from 50 to 100 ng extracted DNA using the PyroMark PCR Kit (QIAGEN 978705). Primers were designed according to the manufacturer’s specifications, and sequences are found in Supplementary Table S2. After confirmation of successful amplification by gel electrophoresis, heteroplasmy levels were analysed by running the PCR product on the Q48 Autoprep workstation along with the appropriate sequencing primer and following the manufacturer’s protocol. Heteroplasmy was then called using the Pyro Mark analysis software and reported as the percentage of mutant base present in the samples.

A PCR-based assay was also used to determine the proportion of wild-type to mutant mtDNA. Specific primers were used to amplify the region where the mtDNA mutation is located, and the dinucleotide substitution in the forward primer creates a restriction site for the restriction enzyme ApaI (New England Biolabs, R0114S), which can cleave the mutant sequence (GGGCCC) when the m.3243 A > G mutation is present. PCR amplification was carried out on a BioRad PCR Thermal Cycler using the following protocol: 1 min denaturation at 94 °C, 1 min annealing at 55 °C, and 45-s extension at 72 °C for 25 cycles. To confirm the presence of the mtDNA mutation, 237-bp PCR samples were made, and the unilateral sample was digested with ApaI at 37 °C for 60 min. These samples were run on a 2% Agarose gel and visualised using GelRed nucleic acid stain using. ApaI digestion produces two units, one of 127 bp and one of 110 bp, which can easily be differentiated from the uncut wild-type unit by gel electrophoresis.

### Quantification of mitochondrial DNA copy numbers in whole organoids using qPCR

The relative mtDNA copy number per cell was quantified by a multiplex Taqman qPCR assay (Bio-rad 4369510) by amplifying *MT-ND1* (mitochondrial encoded gene) and *B2M* (nuclear encoded gene) with a CFX96™ Real-Time PCR Detection System, Bio-Rad, following the protocol described previously^7^. The primers used for template generation of standard curves and the qPCR reaction are described in Supplementary Table S2. The copies per µL of each template were standardised to 1 * 10^10^ and a serial dilution in 1Log_10_ dilution steps, was amplified along with the DNA negative control on each qPCR plate. This was performed in 20 µL reactions in a 96 well-plate, sealed using microplate ‘B’ plate sealers. The reaction mixture was composed of: 5 µL x 5 x Taqman, 0.4 µM of reverse and forward primers, 25–50 ng of DNA template, 0.2 µL MyTaq HS DNA polymerase (Bioline BIO-21112), and PCR-grade autoclaved sterile deionised water (to make up 20 µL reaction mixture). The cycling conditions were as follows: 1) initial denaturation at 95˚C for 3 min, 2) 40 cycles of denaturation at 95˚C for 10 seconds, 3) annealing and extension at 62.5 ˚C for 1 min. The relative mtDNA copy number was calculated using the ΔCt data following the equation: CopyNumber = 2 (2^-ΔCt^), where Delta Ct (ΔCt) equals the sample Ct of the mitochondrial gene (MTND1) subtracted from the sample Ct of the nuclear reference gene (B2M).

### Quantification of mitochondrial DNA copy numbers in neurons and astrocytes using ddPCR

The determine the mtDNA absolute copy numbers by digital droplet PCR, we have used the protocol described by Burr et al^8^. Following Proteinase K treatment, samples were diluted with ddH_2_O 1:125 to obtain the final concentration of 10 cell equivalents per μl. 1 μL cell lysate was combined with locus-specific primers, FAM-labelled MT-ND4 (mtDNA) and HEX-labelled RNAseP (nuclear DNA) probes (Supplementary Table S2), and digital droplet PCR Supermix for probes (no dUTP). All reactions were performed on a QX200 ddPCR system, and each sample was evaluated in technical duplicates or triplicates. Reactions were partitioned into a median of ∼ 10,000 droplets per well using the QX200 droplet generator. Emulsified PCRs were run on a 96-well thermal cycler using cycling conditions identified during the optimisation step (95 °C 10 s; 40 cycles of 94 °C 30 s and 54, 56, or 60 °C 1 s; 98 °C 10 s 4 °C hold). Plates were read and analysed with the QuantaSoft software to assess the number of positive droplets for the gene of interest, reference gene, or neither. mtDNA copy numbers per cell were calculated as follows: MT-ND4 droplet number / RANaseP ^∗^ 2.

### Protein lysate preparation and immunoblotting analysis

Whole organoid slices were lysed in 200 µL of lysis buffer (pH 7.8) (50 mM Tris-HCl- Applichem Biochemica A3452 (pH 7.8) 150 mM NaCl (Merck 1064041000), 1% SDS (Carl Roth CN30.1), and Complete Mini-Roche 11873580001) using a plastic douncer. Then, the samples were centrifuged for 5 min at 4 °C and 5000 × *g*. Protein concentration of the supernatant was determined by BCA assay (Thermo Fisher 23225) (according to the manufacturer’s protocol).

For immunoblot studies, 10 μg protein was used in each case, loaded on a gradient polyacrylamide gel NuPage 4–12% Bis-Tris Protein gels (Thermo Fisher WG1402BOX), and separated for 120 min at 120 V. Following the separation, proteins were transferred to a PVDF membrane (Thermo Fisher IB21001) using the iBlot2 system from Thermo Fisher according to the manufacturer's protocol. Membranes were blocked with 5% Milk prepared in PBS-T for 2 h followed by four washing steps using PBS-T with 0.1% Tween20 (Sigma-Aldrich P7949) (PBS-T). Membranes were incubated with several primary antibodies (Supplementary Table S3) at 4 °C (overnight) and then washed in PBS-T thrice. Horseradish peroxidase conjugated secondary goat anti-rabbit antibody or goat anti-mouse antibody was diluted at 1:25,000 and added to membranes for 1 h. Next, membranes were washed three times in PBS-T for 10 min. By using the enhanced chemiluminescence, horseradish peroxidase substrate (Super-Signal West Pico and Amersham ECL Select Western Blotting Detection Reagent) signals were detected using an Amersham 680.

### Immunocytochemistry

Organoid slices and whole organoids at 30 and 75 days in vitro (DIV) were fixed in 4% paraformaldehyde (PFA) at room temperature under gentle agitation for 45, 25, and 30 min, respectively. Following fixation, samples were washed three times with phosphate-buffered saline (PBS) and incubated in 30% sucrose in PBS for at least 24 h or until they sank. Fixed samples were embedded in Optimal Cutting Temperature (OCT) compound and processed into 12 µm frozen sections for immunostaining.

Sections were encircled with a hydrophobic barrier (Pap Pen), washed in PBS (5 minutes), and permeabilised with 0.5% Triton X-100 (Sigma-Aldrich, t8787) for 10 min. Blocking was performed at room temperature for 1 h in 10% goat or donkey serum (Abcam, D9663, ab7481) in PBS, depending on the species of the secondary antibody. Sections were incubated overnight at 4 °C with primary antibodies diluted in 5% goat or donkey serum in PBS. The following day, sections were washed in PBS and incubated with the corresponding secondary antibody (5% goat or donkey serum in PBS) for 2 h at room temperature. Nuclei were counterstained with 4,6-diamidino-2-phenylindole (DAPI; 0.2 mg/ml), and samples were mounted using ProLong Gold Antifade Mountant (Thermo Fisher Scientific, P36934).

Apoptosis staining was performed using the TUNEL assay (Cell Signalling 25879) according to the manufacturer’s protocol. Samples were co-stained with primary and secondary antibodies as described above.

2D cell cultures were fixed in 4% PFA for 10 minutes at room temperature, followed by permeabilisation with 0.4% Triton X-100 in PBS for 10 minutes. Blocking was performed for 1 hour in 5% bovine serum albumin (BSA) in PBS. Cells were then incubated overnight at 4 °C with primary antibodies diluted in 1% BSA in PBS, followed by secondary antibody incubation for 1 h at room temperature. Samples were mounted using ProLong Gold Antifade Mountant with DAPI.

Formalin-fixed, paraffin-embedded (FFPE) 10-µm human brain tissue sections, provided by Dr. Kieren Allison, were first baked at 60 °C for 2 h to remove the paraffin. Sections were then deparaffinized in xylene (Sigma Aldrich, 214736) for 45 mins, followed by sequential washes in 100, 96, 70, and 50% ethanol (Sigma Aldrich, 32205) (10 minutes each). After rehydration in distilled water, antigen retrieval was performed using 10 mM sodium citrate buffer (Sigma Aldrich, S6639) (0.05% Tween-20, pH 6.0) at 90 °C for 20 min. Following antigen retrieval, sections were washed three times in wash buffer (PBS + 0.3% Triton X-100) and blocked for 2 h in 5% BSA in wash buffer. Samples were incubated overnight at 4 °C with primary antibodies diluted in 5% BSA in PBS. The next day, sections were washed twice in wash buffer, followed by two PBS washes, and incubated with Lipofuscin Autofluorescence Quencher (Biotum, #23007) for 1 min, followed by additional PBS washes. Finally, sections were incubated with secondary antibodies in PBS, washed, and mounted using ProLong Gold Antifade Mountant.

Information about antibodies and dilutions can be found in Supplementary Table S3.

### Image acquisition and processing

Stained organoid microsections and cells were imaged using a 20 ×, 40 ×, or 60 × objective on a Nikon Eclipse Ti-E inverted microscope, equipped with a 4-channel Integrated Laser Engine (ILE-400) and an Andor Dragonfly 200 spinning disk system. The system included two iXon Ultra 888 EMCCD cameras and was operated using Fusion software. Images were acquired at Image frame size was 1024 × 1024 pixels, with automatic Z-step adjustments based on tissue thickness. Whole organoid microsections were imaged using an Opera Phenix Plus High content Screening System from Revvity, using a 40 x water objective. For all experiments, the confocal optical mode was used, and the autofocus was set to two peaks. The binning was set to two, and the camera ROI to 1024 × 1024. A total of 20 stacks were taken with 2 µm distance for each stack. Mitochondrial morphology analysis was conducted using a Zeiss LSM-980 confocal microscope equipped with an Airyscan2 detector to achieve super-resolution imaging. Images were acquired in super-resolution mode, with a bit-depth of 16 bits, covering regions of 28 × 28 µm for cell bodies and 130 × 12 µm for axons.

To ensure complete coverage of cellular structures, Z-stack acquisition was performed with 5 slices at 400 nm intervals for cell bodies and 4 slices at 300 nm intervals for axons. All images were obtained using frame scanning mode. Camera exposure and gain were kept identical while collecting images for each experiment. For illustration purposes, the recommended guidelines were followed and images were only minimally processed in Imaris or Affinity Photo 2 (only brightness and/or contrast) without affecting data presentation. For each sample all channels were uniformly adjusted.

For the neuronal layering analysis of CTIP2 and SATB2 cells in whole organoid microsections post-acquisition image analysis was performed using StarDist^9^ (https://github.com/stardist/stardist) in QuPath^10^ (0.5.12.; https://qupath.github.io/). Raw imaging data acquired from the Opera Phenix high-content screening system was exported from Harmony software, then Z-projected and stitched at full resolution using Fiji (ImageJ) with the ‘Operetta Importer’ and the ‘Grid/Collection Stitching’ plugin. Images were imported as maximum intensity projections with a down-sampling factor of 1 to preserve high resolution. Illumination correction for the DAPI channel was applied in Fiji, setting the central intensity to 1 with increasing values toward the periphery to allow for a better nuclear segmentation. Nuclei segmentation was conducted in QuPath using a pre-trained StarDist model optimised for whole-slide image analysis (https://github.com/tpecot/WholeSlideImageAnalysisWithQuPath)^11–13^. The QuPath StarDist extension was employed to detect nuclear boundaries based on DAPI staining, with segmentation parameters adjusted to a detection threshold of 0.5, a pixel size of 0.5 µm, and a minimum nucleus area of 30 µm² to exclude debris. Following segmentation, nuclei were evaluated for CTIP2 and SATB2 expression based on manually set fluorescence intensity thresholds, which were kept constant across all images. The percentage of CTIP2-positive or SATB2-positive nuclei relative to the total DAPI-positive nuclei was then calculated.

For the human tissue, the MAP2-positive area was quantified using QuPath (v0.5.1) with a tile-based approach. For each section, the tissue area was manually annotated to exclude background and non-tissue regions. Annotated regions were subdivided into fixed-size square tiles (10 µm × 10 µm). Mean fluorescence intensity in the MAP2 (green) channel was calculated for each tile at full resolution (downsample 1). Tiles were classified as MAP2-positive using a fixed intensity threshold, which was determined empirically from background signal and applied uniformly across all images. MAP2-positive area was expressed as the percentage of MAP2-positive tiles relative to the total number of tiles within each ROI, thereby normalising for differences in ROI size.

For mitochondrial morphology, post-acquisition image analysis was carried out using Fiji (ImageJ) software, where background subtraction with a 50-pixel radius, despeckle filtering, and Gaussian blur with a 1-pixel radius were applied to enhance image quality. Mitochondrial segmentation within the cell body and axonal regions was performed based on TOM20 intensity thresholding, and the resulting binary masks were subjected to particle analysis to extract quantitative morphological parameters. These parameters included mitochondrial area, which measured the total mitochondrial coverage within the analysed regions; perimeter, representing the total boundary length of individual mitochondria to assess shape complexity; form factor, a measure of mitochondrial shape complexity calculated as the ratio of perimeter squared to area, with values approaching 1 indicating a more circular shape and lower values suggesting elongated mitochondria; and aspect ratio, defined as the ratio of the major to minor axis of mitochondria, providing insights into their elongation and branching behaviour. In addition, mitochondrial density was calculated as the number of mitochondria per micrometre of axonal length to provide a quantitative assessment of mitochondrial distribution along the axon.

Axonal swellings were quantified using CellProfiller (version 4.2.8; https://cellprofiler.org) with a custom image analysis pipeline. To enhance the visibility of the swellings, images were first pre-processed using the Smooth module with the Smooth Keeping Edges method, applying an edge intensity difference threshold of 0.05. The swellings were then identified using the IdentifyPrimaryObjects module, with a diameter range set between 2–25 pixels. An adaptive two-class Otsu thresholding method was applied. The objects were subsequently analysed using the MeasureObjectSizeShape module to extract morphological measurements. Finally, identified objects were filtered using the FilterObjects module, retaining those with a form factor between 0.7 and 1 and an area between 6–170 pixels to exclude artifacts and background noise. Thee areas of interest from two different batches were analysed for each sample.

### scRNA sequencing pipeline and analysis

Reads were aligned to the GRCh38 human genome using Cell Ranger (version 7.0.1) that additionally performs filtering, barcode and UMI counting to obtain barcode and feature matrices. CellRanger detected 88.4% fraction reads on average per cell (ranging from 83.7% to 92.0%) and 1443-4368 (3383 on average) median genes per cell. Bias from false cell discovery due to potential ambient RNA contamination/low UMI counts was not eliminated in any of the samples. A conda environment has been created to install all required software, among which R and Satija’s Seurat R package-version 4.0/5.0.0^14^, R version 4.2.2.

To remove low quality cells and ensure high-quality data, cells with more than 20% of mitochondrial or ribosomal reads have been removed; the ratio between the log (number of genes) and log (number of molecules) has been evaluated for each cell; only cells that have a high score of such value ( >0.8) and a high number of detected molecules ( > 1000) and a detected number of genes between 800 and 10000 have been kept. To remove low detected genes, those that are expressed in less than 10 cells have been removed. In addition, long non-coding genes, antisense transcripts and pseudogenes have been removed from the analysis. The resulting dataset has 46725 genes. Normalisation and integration have been performed with sctranform, an R package pipeline, using the top 3000 features as identified by the SelectIntegrationFeatures function, to perform the integration step. To reduce the dimensionality of the dataset, a PCA and Uniform Manifold Approximation and Projection (UMAP) have been performed, the latter using the first 20 dimensions of the dataset, as determined with the Elbow method. The cell cycle stage has been determined using the CellCycleScoring function of the Seurat package.

To identify the cell clusters, we have employed the FindClusters method, selecting the Louvain algorithm with multi-level refinement, setting the clustering resolution to 0.185. This led to the identification on one cluster of stressed cells that was removed from further analysis. The final size of the dataset is of 119.591 cells. Clustering has been performed a second time, setting the clustering parameter to 0.18. To annotate the cell types, we calculated the top 50 marker genes per cluster, using the FindAllMarkers Seurat function, using the Wilcoxon test and selecting only the positively expressed genes with a logarithmic fold change greater than 0.6 and a minimum percentage of cells in each cluster that express the gene of 0.6.

Cell maturity analysis was achieved by independently projecting data from our 100DIV organoids onto one scRNA-seq data set from foetal brain^15^ using scmap (v1.20.2) software^16^. We employed scmapCluster function of the scmap R package (‘scmapCluster()’ threshold = 0)).

Monocle3 (v1.3.1) was employed for trajectory reconstruction, leaving the default parameters for the order_cells function and choosing RG as the starting cell type.

Transcription factory activity analysis was performed using pyscenic (v0.12.0), Python version 3.8.13, following the standard pipeline (GRNboost2, AUCell), leaving the default parameter values.

The Wilcoxon test was used to compare the distribution of continuous variables between groups, for example, gene expression. Pearson’s chi-squared test was used to compare distributions of categorical data. *P*-values have been adjusted using the Benjamini-Hochberg procedure.

To identify genes that change in expression across conditions, we performed a differential expression analysis. Genes were identified with the FindMarkers function, setting the minimum number of cells expressing the gene to 3. Genes are considered differentially expressed (DEG) if the adjusted *p*-values is smaller than 0.05 and the logarithmic fold change is greater or smaller than 0.5. Overlap of the significant terms with the Mitocarta database (version 3.0) has been performed.

Pathway enrichment analysis was performed using ClusterProfiler (v4.6.2) on gene sets identified by the differential expression analysis. Following are the parameters set for the enrichGo function: org.Hs.eg.db (v3.16.0) as reference organism database, *p*-values have been corrected with the “BH” method, *p*-value and q-value cutoffs are set to 0.05, and the “MF”, “BP”, “CC” ontologies have been selected.

### Statistics

Sample sizes using iPSC lines and organoids were estimated from previous studies^17,19^. For this study, we derived 5 independent batches of organoids from each iPSC line. For our experiments, organoids used are from different batches unless specified. For some experiments, such as heteroplasmy measurements we wanted to determine inter and intra batch variation. Experiments were repeated three times (or two times absolute quantification of mtDNA in sorted neurons and mitochondrial morphology in 2D neurons), which included at least three independent biological replicates, and all had similar results. For organoids experiments at least three independent biological replicates were used per group for all statistical analyses in biological experiments. For Seahorse experiments, all assays were performed three times to confirm the reproducibility of the observed patterns. The reported *n* = 5 refers to five independent organoids run on the same plate to minimise inter-plate variability, which is a standard practice for Seahorse assays. Seahorse experiments on 2D neuronal cultures were conducted using cells obtained from three independent differentiation batches (*n* = 3).

Errors bars displayed on graphs represent the mean +/− standard error of the mean (S.E.M.). The number of experiments and cells analysed is shown in the corresponding paragraph in material and methods. Statistical significance was analysed using the tests described in the corresponding figure legends. All statistical analyses and super-plots from the experimental data were performed using GraphPad (version 10), while scRNA-seq were performed using R. Data distributions were assessed for normality in GraphPad Prism (Shapiro-Wilk and D’Agostino-Pearson tests). When normality assumptions were not met, non-parametric tests were applied. All statistical tests were two-tailed unless otherwise stated. Comparisons between two independent groups were performed using the Mann-Whitney test, while comparisons among more than two groups were performed using the Kruskal-Wallis test followed by Dunn’s multiple comparisons test. For parametric analyses involving more than two groups, one-way ANOVA with Tukey’s or Dunnett’s multiple comparisons test was used. Unless stated otherwise, statistical significance was accepted at *P*  <  0.05, and the exact *P* values are included in the graphs. Where no significant differences were detected among groups, the overall ANOVA *p*-value is reported, and non-significant values are not shown in the plot.

### Reporting summary

Further information on research design is available in the Nature Portfolio Reporting Summary linked to this article.

## Supplementary information

Supplementary Infromation

Description of Additional Supplementary Files

Supplementary Data 1-5

Reporting Summary

Transparent Peer Review file

## Source data

Source Data

## Data Availability

The single-cell RNA sequencing data generated in this study have been deposited in the NCBI Gene Expression Omnibus (GEO) under accession number GSE324301. Source data, including high-resolution scans of all blots presented in this paper, are provided as a Source Data file. All other data supporting the findings of this study are available within the paper and its Supplementary Information. This paper does not report any original code Source data are provided in this paper.
