# Supplementary material for: Mitochondrial DNA heteroplasmy drives cortical neuronal disturbances in human organoids harbouring the common m.3243A>G mutation
Source: Nat Commun. 2026 Jun 21;17:7840. doi: 10.1038/s41467-026-74103-y (PMC13438594; doi:10.1038/s41467-026-74103-y)
Supplement: Supplementary file 1 [file 41467_2026_74103_MOESM2_ESM.pdf]

Description of Additional Supplementary Files

Supplementary Data 1

Top 50 marker genes per cluster identified from the merged scRNA-seq dataset. Marker genes were identified using a two-sided Wilcoxon rank-sum test, selecting positively expressed genes with log fold change > 0.6 and minimum cell expression percentage of 0.6 per cluster.

Supplementary Data 2

Differentially expressed gene transcripts in DLN5B comparing high versus low heteroplasmy conditions by scRNA-seq. Statistical analysis: two-sided Wilcoxon rank-sum test; p-values adjusted using the Benjamini-Hochberg procedure; DEGs defined as adjusted p-value < 0.05 and log fold change > 0.5.

Supplementary Data 3

Differentially expressed genes in DLN5B comparing high versus low heteroplasmy conditions by scRNA-seq. Statistical analysis: two-sided Wilcoxon rank-sum test; p-values adjusted using the Benjamini-Hochberg procedure; DEGs defined as adjusted p-value < 0.05 and log fold change > 0.5.

Supplementary Data 4

Differentially expressed genes in high-heteroplasmy DLN5B neurons compared to ULN. Statistical analysis: two-sided Wilcoxon rank-sum test; p-values adjusted using the Benjamini-Hochberg procedure; DEGs defined as adjusted p-value < 0.05 and log fold change > 0.5.

Supplementary Data 5

Differentially expressed genes in control-heteroplasmy DLN5B neurons compared to ULN. Statistical analysis: two-sided Wilcoxon rank-sum test; p-values adjusted using the Benjamini-Hochberg procedure; DEGs defined as adjusted p-value < 0.05 and log fold change > 0.5.
