# Supplementary material for: Mitochondrial DNA heteroplasmy drives cortical neuronal disturbances in human organoids harbouring the common m.3243A>G mutation
Source: Nat Commun. 2026 Jun 21;17:7840. doi: 10.1038/s41467-026-74103-y (PMC13438594; doi:10.1038/s41467-026-74103-y)
Supplement: Supplementary file 2 [file 41467_2026_74103_MOESM4_ESM.pdf]

nature portfolio

Corresponding author(s): Denisa Hathazi, Andras Lakatos and Rita Horvath

Last updated by author(s): 28/01/26

Reporting Summary

Nature Portfolio wishes to improve the reproducibility of the work that we publish. This form provides structure for consistency and transparency in reporting. For further information on Nature Portfolio policies, see our Editorial Policies and the Editorial Policy Checklist.

Statistics

For all statistical analyses, confirm that the following items are present in the figure legend, table legend, main text, or Methods section.

n/a	Confirmed	
☐	☒	The exact sample size (*n*) for each experimental group/condition, given as a discrete number and unit of measurement
☐	☒	A statement on whether measurements were taken from distinct samples or whether the same sample was measured repeatedly
☐	☒	The statistical test(s) used AND whether they are one- or two-sided<br>*Only common tests should be described solely by name; describe more complex techniques in the Methods section.*
☐	☒	A description of all covariates tested
☐	☒	A description of any assumptions or corrections, such as tests of normality and adjustment for multiple comparisons
☐	☒	A full description of the statistical parameters including central tendency (e.g. means) or other basic estimates (e.g. regression coefficient) AND variation (e.g. standard deviation) or associated estimates of uncertainty (e.g. confidence intervals)
☐	☒	For null hypothesis testing, the test statistic (e.g. *F*, *t*, *r*) with confidence intervals, effect sizes, degrees of freedom and *P* value noted<br>*Give P values as exact values whenever suitable.*
☒	☐	For Bayesian analysis, information on the choice of priors and Markov chain Monte Carlo settings
☒	☐	For hierarchical and complex designs, identification of the appropriate level for tests and full reporting of outcomes
☒	☐	Estimates of effect sizes (e.g. Cohen's *d*, Pearson's *r*), indicating how they were calculated

Our web collection on statistics for biologists contains articles on many of the points above.

Software and code

Policy information about availability of computer code

Data collection	PyroMark Q48 software, Wave Software, Zeiss Zen, FACSDiva software, Harmony software, Fusion software, CFX Manager software, LAS X software, QuantaSoft Pro software, Omega Control software
Data analysis	GraphPad (version 10), Cell Ranger (version 7.0.1), R and Satija's Seurat R package-version 4.0/5.0.0, R version 4.2.2, scmap (v1.20.2) software, pyscenic (v0.12.0), python version 3.8.13, ClusterProfiler (v4.6.2), enrichGo function: org.Hs.eg.db (v3.16.0), Adobe Illustrator 2024, Affinity Photo 2

For manuscripts utilizing custom algorithms or software that are central to the research but not yet described in published literature, software must be made available to editors and reviewers. We strongly encourage code deposition in a community repository (e.g. GitHub). See the Nature Portfolio guidelines for submitting code & software for further information.

Data

Policy information about availability of data

All manuscripts must include a data availability statement. This statement should provide the following information, where applicable:

- Accession codes, unique identifiers, or web links for publicly available datasets
- A description of any restrictions on data availability
- For clinical datasets or third party data, please ensure that the statement adheres to our policy

The single-cell RNA sequencing data have been deposited in the NCBI Gene Expression Omnibus (GEO), an approved publicly accessible repository, under accession number GSE324301

-figures with associated data (see source data and uncropped blots)
-data is also always available from the corresponding authors

Research involving human participants, their data, or biological material

Policy information about studies with human participants or human data. See also policy information about sex, gender (identity/presentation), and sexual orientation and race, ethnicity and racism.

Reporting on sex and gender	iPSC lines were derived from two female donors. Sex was not used as a variable in the analysis, as the study focused on cell-type-specific metabolic responses and included only female-derived lines.
Reporting on race, ethnicity, or other socially relevant groupings	Race and ethnicity information was available for one donor (Caucasian) and not reported for the second donor. Race/ethnicity was not used as a variable in the analysis, as this study focused on cell-type-specific metabolic phenotypes in iPSC-derived cells.
Population characteristics	PSC lines were generated from two female donors with mitochondrial disease. Donors differed in age at biopsy and clinical presentation but shared a common pathogenic mitochondrial DNA mutation. The study was not designed to assess population-level variation.
Recruitment	Donors were recruited through specialist mitochondrial disease clinics, and skin biopsies were obtained with informed consent for research use. Samples were collected as part of clinically approved protocols and subsequently reprogrammed into iPSCs.
Ethics oversight	REC ID: 13/YH/0310, Yorkshire & Humber – Bradford Leeds Research Ethics Committee

Note that full information on the approval of the study protocol must also be provided in the manuscript.

Field-specific reporting

Please select the one below that is the best fit for your research. If you are not sure, read the appropriate sections before making your selection.

☒ Life sciences ☐ Behavioural & social sciences ☐ Ecological, evolutionary & environmental sciences

For a reference copy of the document with all sections, see nature.com/documents/nr-reporting-summary-flat.pdf

Life sciences study design

All studies must disclose on these points even when the disclosure is negative.

Sample size	Statistical test details are included in source data. No sample size calculation was performed. The sample sizes using hiPSC lines and organoids were estimated from previously performed experiments (eg. Szebényi et al., Nature Neurosciences, 2021; Giandomenico et al., Nature Neuroscience 2019)
Data exclusions	No acquired data was excluded from the statistical analyses. (A few damaged sections/slices were not used for data collection).
Replication	Experiments were repeated three times (or two times for the sorting of neurons and astrocytes) and included at least three biological replicates (organoids or cells grown from separate indipendent batches and/or cell lines)
Randomization	The in vitro experiments were not fully randomized. For cell-based experiments with control and treatment conditions, samples were allocated using independent biological replicates derived from different cell lines and/or separate differentiation batches. Batches of cells were randomly selected from each cell line group for inclusion in experimental comparisons. Organoid-derived samples generated from the same organoid were used only in separate experiments.
Blinding	Data analysis was performed with partial blinding. Where feasible, sample identity was masked during quantification and revealed only after analysis was completed.

Reporting for specific materials, systems and methods

We require information from authors about some types of materials, experimental systems and methods used in many studies. Here, indicate whether each material, system or method listed is relevant to your study. If you are not sure if a list item applies to your research, read the appropriate section before selecting a response.

Materials & experimental systems

n/a	Involved in the study
☐	☒ Antibodies
☐	☒ Eukaryotic cell lines
☒	☐ Palaeontology and archaeology
☒	☐ Animals and other organisms
☒	☐ Clinical data
☒	☐ Dual use research of concern
☒	☐ Plants

Methods

n/a	Involved in the study
☒	☐ ChIP-seq
☐	☒ Flow cytometry
☒	☐ MRI-based neuroimaging

Antibodies

Antibodies used	All details of antibodies used are found in Table S3
Validation	Antibodies were validated by the manufacturers for the applications used and further assessed by detection of bands at the expected molecular weight (for immunoblotting) and by appropriate cellular localisation in immunofluorescence experiments.

Eukaryotic cell lines

Policy information about cell lines and Sex and Gender in Research

Cell line source(s)	All cell lines sources are in Table S1 and Materials and Methods
Authentication	We checked the mutations via pyrosequencing (Figure 1) and also via by PCR-RFLP where a 528 bp PCR fragment is digested with ApaI (Figure S1)
Mycoplasma contamination	Media from cell lines were randomly tested for Mycoplasma in our laboratory, and there has been no evidence for contamination by Mycoplasma.
Commonly misidentified lines (See ICLAC register)	No commonly misidentified cell lines were used.

Plants

Seed stocks	*Report on the source of all seed stocks or other plant material used. If applicable, state the seed stock centre and catalogue number. If plant specimens were collected from the field, describe the collection location, date and sampling procedures.*
Novel plant genotypes	*Describe the methods by which all novel plant genotypes were produced. This includes those generated by transgenic approaches, gene editing, chemical/radiation-based mutagenesis and hybridization. For transgenic lines, describe the transformation method, the number of independent lines analyzed and the generation upon which experiments were performed. For gene-edited lines, describe the editor used, the endogenous sequence targeted for editing, the targeting guide RNA sequence (if applicable) and how the editor was applied.*
Authentication	*Describe any authentication procedures for each seed stock used or novel genotype generated. Describe any experiments used to assess the effect of a mutation and, where applicable, how potential secondary effects (e.g. second site T-DNA insertions, mosiacism, off-target gene editing) were examined.*

Flow Cytometry

Plots

Confirm that:

- ☒ The axis labels state the marker and fluorochrome used (e.g. CD4-FITC).
- ☒ The axis scales are clearly visible. Include numbers along axes only for bottom left plot of group (a 'group' is an analysis of identical markers).
- ☒ All plots are contour plots with outliers or pseudocolor plots.
- ☒ A numerical value for number of cells or percentage (with statistics) is provided.

Methodology

Sample preparation	Samples were prepared as described by Yu Nie, Scienece Advances 2025
Instrument	FACSAria™ Flow Cytometer
Software	FACSDiva software (BD Biosciences)

Cell population abundance	Cell population abundance was not used as a quantitative outcome in the study. Flow cytometry was performed primarily to isolate astroglial and neuronal populations based on marker expression. Percentages of marker-positive cells are shown in plots for illustrative purposes only and were not included in downstream statistical analyses.
Gating strategy	Gates were set using an isotype control (mouse IgG1). Events were first filtered to exclude debris and doublets, and viable singlet cells were then classified based on marker expression. HEPACAM-positive cells were identified as astroglia and L1CAM-positive cells as neurons.

☐ Tick this box to confirm that a figure exemplifying the gating strategy is provided in the Supplementary Information.
