# Supplementary material for: Mitochondrial DNA heteroplasmy drives cortical neuronal disturbances in human organoids harbouring the common m.3243A>G mutation
Source: Nat Commun. 2026 Jun 21;17:7840. doi: 10.1038/s41467-026-74103-y (PMC13438594; doi:10.1038/s41467-026-74103-y)
Supplement: Supplementary file 3 [file 41467_2026_74103_MOESM5_ESM.pdf]

nature portfolio

# Peer Review File

# Mitochondrial DNA heteroplasmy drives cortical neuronal disturbances in human organoids harbouring the common m.3243A>G mutation

Corresponding Author: Dr Denisa Hathazi

**This file contains all reviewer reports in order by version, followed by all author rebuttals in order by version.**

Version 0:

Reviewer comments:

Reviewer #2

(Remarks to the Author)
The study by Hathazi et al. presents a novel and well-executed study investigating the impact of pathogenic mitochondrial heteroplasmy on neuronal subpopulations using human cerebral organoids and 2D monocultures. The study is methodologically sound, combining single-cell transcriptomics, confocal imaging, and seahorse metabolic profiling. The authors demonstrate that high levels of pathogenic mtDNA heteroplasmy lead to specific deficits in deep-layer cortical neurons, including altered mitochondrial morphology and axon integrity, as well as increased cell death. The use of brain organoids with controlled heteroplasmy levels represents an important step forward in mitochondrial disease research, addressing the growing need for physiologically relevant, complex, multicellular models.

A major strength of the study is the use of isogenic iPSCs and thereof derived organoids that differ only in their mtDNA heteroplasmy levels (low, medium, and high). The authors demonstrate that organoids largely maintain their heteroplasmy levels over time. While comparisons across two patient-derived lines are made (and both patient-derived high heteroplasmy organoids show the same reduction in deep layer neurons), some differences are noted between the lines. This is expected though, given differences in age at biopsy and clinical presentation. It would be helpful if the authors could briefly elaborate on these patient differences in the main text to aid interpretation.

The authors have not included functional assays beyond seahorse profiling, such as electrophysiology, which limits the interpretation of how mitochondrial deficits affect neuronal activity. Furthermore, seahorse measurements were performed only at DIV100, whereas for instance a compensatory increase in mtDNA copy number was noted at DIV50, which drops at DIV100 in high heteroplasmy organoids. I wonder whether the organoids enter a hypermetabolic state over time, followed by a functional decline at later stages. More comprehensive seahorse profiling, including earlier time points and, ideally, at the cell type level, would strengthen these findings. While not critical for the current study, this would be a valuable direction for future work, particularly given that organoids model a developing, rather than mature, brain.

The inclusion of post-mortem MELAS brain tissue adds translational value by recapitulating the selective loss of deep-layer neurons seen in organoids. However, this comparison is based on a single patient, and as far as I can see, a single tissue section with limited detail on how neuronal loss was properly quantified. Without staining for CTIP2, proper quantification of neuronal loss, and with no specific heteroplasmy percentage reported for the brain tissue, these data remain suggestive but not conclusive.

Specific comments and suggestions:
- Figure 2: The immunofluorescence panels lack logical ordering by heteroplasmy level. A consistent layout across both patient lines would improve clarity. Also, SATB2 appears visually reduced at high heteroplasmy in Fig. 2D, but this is not reflected in the quantifications. it would be helpful if the authors could provide representative examples of their image preprocessing. Given the central importance of these findings, showing what signal is retained or excluded during quantification would strengthen confidence in the data.
- Figure 3 (Seahorse assay): To support the claim of a metabolic shift toward glycolysis, ATP production rates should ideally be calculated (see https://doi.org/10.15252/embr.202256380 or https://doi.org/10.1074/jbc.M116.774471). As ECAR reflects both glycolytic acidification from lactate and CO2 hydration coming from the TCA, a lower OCR/ECAR ratio alone is not

sufficient to infer a glycolytic shift. The observed reduction in respiration in high-heteroplasmy organoids may contribute to this ratio, suggesting a potential mixed effect rather than a pure increase in glycolytic flux.
- Is there a reason why the authors have only looked at complexes I, II, IV, and V, but ignored complex III in their profiling? (see figures 3 and 6)
- I appreciate that the authors quantified mtDNA copy numbers in neurons and astrocytes, though it's a bit unclear why astrocytes were the only glial population included. Expanding this analysis to other glial cell types in future studies would be great, as glial cells are often underappreciated. I look forward to more cell-type–specific insights in this area.
- Line 303: The statement that "these findings well reflect the loss of CTIP2$^{+}$ deep layer neurons found in organoids" is misleading; CTIP2 staining was not performed in the post-mortem tissue, and the loss of DLNs was not quantified. With an n of 1, this could reflect tissue quality rather than biology.
- Figure S5B (GFAP staining): The image is difficult to interpret without a control comparison.
- Figure 6 F-H: Visual inspection suggests the data are not normally distributed. The authors report using non-parametric tests when assumptions are not met (line 873), but Anova is still used in this figure. Please clarify whether assumptions (normality, equal variance) were tested (e.g., via Q-Q plots or residual analysis), and consider using a non-parametric alternative if necessary.
- Seahorse methods: FCCP concentration is not reported. Maximal respiration/spare capacity cannot be interpreted without this information, and FCCP dose should ideally be titrated. Also, how was total DNA quantified with Hoechst, was it pixel-based or a nuclei count? Also, what do the authors mean with "ATP production"? Is it ATP-linked respiration, i.e. baseline - post-oligo? Again, calculating ATP production rates for OxPhos and glycolysis would help with the interpretation.

Minor corrections:
- Fig1C batches 1 and 3 have the same color-code
- Panel Fig 2G has plus-symbols, probably from text formatting
- The legend in Fig 2H is cut off
- Fig. S3C where is the 69% heteroplasmy coming from? --> please check for consistency throughout the manuscript
- Figure 3E/F: The legend is missing detail about the color code. It's unclear what the green and blue represent, especially as both colors appear within each patient group.
- Figure S4B (line 298): Should be labeled as Figure S5B. Please double-check figure references throughout.

Reviewer #3

(Remarks to the Author)
This study aims to establish and characterise a cellular model of heteroplasmic mutation of the mitochondrial MT-TL1 gene (m.3243A>G) using human patient iPSC-derived cerebral organoids. Through single-cell transcriptome analyses and comparisons of different heteroplasmy levels in these iPSC-derived cerebral organoids, the authors concluded that the m.3243A>G variant does not lead to transcriptional changes associated with cell differentiation, except for a reduction in the proportions of DL cells. Immunostaining analyses confirmed the reduction of CTIP2+, DL-identity marker, cells in organoids with high levels of heteroplasmy. Additionally, the authors found that reduced mitochondrial OXPHOS proteins and reduced mitochondrial oxygen consumption rate were associated with a high level of heteroplasmy. Furthermore, the author observed an increased number of TUNEL-positive cells in CTIP2+ cells, which may explain the reduced proportion of DL neurons in organoids with high heteroplasmy, along with axonal structural abnormalities. The authors addressed important and timely questions to gain a deeper understanding of the pathogenesis associated with MT-TL1 gene mutations. Their findings have the potential to enhance our understanding of how mitochondrial heteroplasmy impacts cellular function. However, to support the conclusion presented in the manuscript, the data in several sections (as detailed below) would need substantial strengthening, likely involving new experiments.

Major points:
1. Cell-type specific vulnerability:
The author demonstrated a reduction in deep layer (DL) and CTIP2+ neurons in high heteroplasmy organoids through transcriptome analysis (Figure 1E) and immunostaining (Figures 2B-E). Based on the increased proportion of TUNEL+ cells among CTIP2+ neurons (Figures 5B,C), the author concludes that high heteroplasmy promotes apoptosis in deep layer neurons, leading to a specific loss of DL neurons in these organoids.

To strengthen the author's conclusion and better interpret the results of Figure 2E, it would be beneficial to compare neuronal populations across organoids with varying levels of heteroplasmy. This comparison could involve immunostaining for a pan-neuronal marker, such as NeuroD2.

Furthermore, what is the impact of high heteroplasmy on the proportion of TUNEL+ cells in other cell types, such as upper layer (UL) neurons and glia? It is essential to determine whether the effects are specific to DL neurons.

Additionally, another possibility, such as the reduction in the production of DL neurons caused by the m.3243 A>G mutation, has not been explored.

2. Links between levels of heteroplasmy m.3243 A>G and mitochondrial defects:
Several reports indicate differences in mitochondrial activity and metabolism between cortical progenitors and neurons, with neurons exhibiting higher oxidative phosphorylation (OXPHOS) activity than their progenitors. To understand the impact of high heteroplasmy on neuronal mitochondrial functions, it is important to include information about the ratio of neurons to progenitors in the experiments shown in Figure 3 (WB and Seahorse assays). Additionally, conducting the Seahorse assay with isolated neurons to measure oxidative activity could further strengthen the authors' conclusions. In this line, the authors

highlighted the differences in mtDNA copy number between whole organoid-derived cells and isolated neurons in Figures 3E and 3F.
Furthermore, analysing the expression levels of other proteins coded by mitochondrial DNA would help generalise the effects of high levels of heteroplasmy of m.3243 A>G mutation.

3. MELAS patient phenotype:
Identifying the conserved features between the organoid model and MELAS patients is crucial for accurately replicating the pathology in the organoid model. In this context, evaluating the proportion of SATB2+ and CTIP2+ neurons, as well as the presence of axonal abnormalities in MELAS patient tissue is essential.

Minor points:
4. Why do the “S7 DEGS DLN5B vs ULN high h” contain the same genes, such as CDH7, GRIA2, and DPP10, in both UP-regulated and DOWN-regulated groups? Is this associated with the Figure 4J (right) result? If so, why are MT-DN2, ND3, and ND5 not listed in the S7 table?

5. Typos:
Line 93; Line 226 Figure 3SD; Line 231 Figure 3SE, Line 295 Figure S4A, Line 298 Figure S4B

6. Figure S5G right top, images are inverted between TOM20+SMI-321 and TOM20.

7. Figure 6G, There is “A.” within the figure

8. Figure 5F, what does the blue colour indicate?

9. Why is PLAGL1 missing from the list in Figure S4B?

10. Why is high heteroplasmy related to a TF, STAT4, categorised as “absent” in the HIGH group in Figure S4B?

Version 1:

Reviewer comments:

Reviewer #2

(Remarks to the Author)
Thank you for the detailed and thoughtful responses to all of my comments. Overall, my concerns have been adequately addressed, and the revisions substantially strengthen the manuscript.

I have one remaining minor point regarding the newly added description of OXPHOS subunits on page 7 of the main text (i.e. point 8 in the rebuttal letter). Specifically, the statement that MT-ATP6 “remained unchanged” across all organoids does not appear accurate based on the quantification, where MT-ATP6 is reduced by ~40% in the high-heteroplasmy condition. I recommend revising this wording (e.g. “moderately reduced” instead of "unchanged") and briefly acknowledging that the concordant reduction across mtDNA-encoded markers (MT-CO1, MT-CO2) could reflect a genome-level effect on mtDNA-encoded protein abundance (independent of copy number), rather than a strictly complex-specific effect.

Reviewer #3

(Remarks to the Author)
The authors addressed all reviewers' points, and their results support their conclusion. This work is timely and contributes to the broad field of neurodevelopmental biology.

**Open Access** This Peer Review File is licensed under a Creative Commons Attribution 4.0 International License, which permits use, sharing, adaptation, distribution and reproduction in any medium or format, as long as you give appropriate credit to the original author(s) and the source, provide a link to the Creative Commons license, and indicate if changes were made.
In cases where reviewers are anonymous, credit should be given to 'Anonymous Referee' and the source.
The images or other third party material in this Peer Review File are included in the article's Creative Commons license, unless indicated otherwise in a credit line to the material. If material is not included in the article's Creative Commons license and your intended use is not permitted by statutory regulation or exceeds the permitted use, you will need to obtain permission directly from the copyright holder.
To view a copy of this license, visit https://creativecommons.org/licenses/by/4.0/

Dear Editor and Reviewers,

We thank you for the careful and constructive evaluation of our manuscript entitled "**Mitochondrial DNA heteroplasmy drives cortical neuronal disturbances in human organoids harbouring the common m.3243A>G mutation**".

We have revised the paper based on the comments of the reviewers`. We have conducted additional experiments and made significant revisions to the manuscript text to address the specific concerns raised. We believe that this revised version strengthens the message of our paper. We have also added two additional authors that have contributed to the revision of this manuscript. Please find our point-by-point response below in blue.

Thank you very much for the reconsideration of our manuscript.

Sincerely,

Denisa Hathazi, Andras Lakatos and Rita Horvath

**REVIEWER COMMENTS**

**Reviewer #2** (Remarks to the Author):

1. The study by Hathazi et al. presents a novel and well-executed study investigating the impact of pathogenic mitochondrial heteroplasmy on neuronal subpopulations using human cerebral organoids and 2D monocultures. The study is methodologically sound, combining single-cell transcriptomics, confocal imaging, and seahorse metabolic profiling. The authors demonstrate that high levels of pathogenic mtDNA heteroplasmy lead to specific deficits in deep-layer cortical neurons, including altered mitochondrial morphology and axon integrity, as well as increased cell death. The use of brain organoids with controlled heteroplasmy levels represents an important step forward in mitochondrial disease research, addressing the growing need for physiologically relevant, complex, multicellular models.

We thank the reviewer for their thoughtful and positive assessment of our study.

2. A major strength of the study is the use of isogenic iPSCs and thereof derived organoids that differ only in their mtDNA heteroplasmy levels (low, medium, and high). The authors demonstrate that organoids largely maintain their heteroplasmy levels over time. While comparisons across two patient-derived lines are made (and both patient-derived high heteroplasmy organoids show the same reduction in deep layer neurons), some differences are noted between the lines. This is expected though, given differences in age at biopsy and clinical presentation. It would be helpful if the authors could briefly elaborate on these patient differences in the main text to aid interpretation.

Patient 1, is a 73-year-old female patient who presented with maternally inherited diabetes, deafness (MIDD) and pigmentary retinopathy, while patient 2 is a 17year-old female with mitochondrial encephalomyopathy, lactic acidosis, and stroke-like episodes (MELAS), hearing loss and diabetes.

We have now added a brief description of the clinical presentations to the main text in the results section on page 4:

'We used iPSC lines derived from two patients carrying the m.3243A>G mutation (Figure 1A), clinically presenting with MIDD and pigmentary retinopathy (Patient 1) and MELAS, hearing loss and diabetes (Patient 2). The clinical data of these patients are summarized in Table S1. From each line, we generated low, medium, and high heteroplasmy clones to model controlled heteroplasmy ranges independent of the original bulk heteroplasmy.'

3. The authors have not included functional assays beyond seahorse profiling, such as electrophysiology, which limits the interpretation of how mitochondrial deficits affect neuronal activity. Furthermore, seahorse measurements were performed only at DIV100, whereas for instance a compensatory increase in mtDNA copy number was noted at DIV50, which drops at DIV100 in high heteroplasmy organoids. I wonder whether the organoids enter a hypermetabolic state over time, followed by a functional decline at later stages. More comprehensive seahorse profiling, including earlier time points and, ideally, at the cell type level, would strengthen these findings. While not critical for the current study, this would be a valuable direction for future work, particularly given that organoids model a developing, rather than mature, brain.

We thank the reviewer for this constructive comment. We agree that functional assays such as electrophysiological recordings would provide valuable insight into how mitochondrial

dysfunction affects neuronal network activity. In the present study, we did not include MEA recordings however we have recently started the recording of MEA in 200DIV organoids with high heteroplasmy and respective isogenic controls and we included these pilot data only for the reviewer. (See figure below). MEAs were recorded using Multi Channel Systems, MEA2100. Six-minute-long recordings of spontaneous activity at 37 °C ($n = 9$ ALI-COs) were acquired and exported to Matlab (MathWorks) for analysis. Our preliminary data show a non-significant reduction in mean firing rate and a significant increase in interspike interval in high heteroplasmic organoids, consistent with impaired network activity and network asynchrony. As these findings require further validations, we plan to extend this work to a full electrophysiological analysis in a dedicated follow up study.

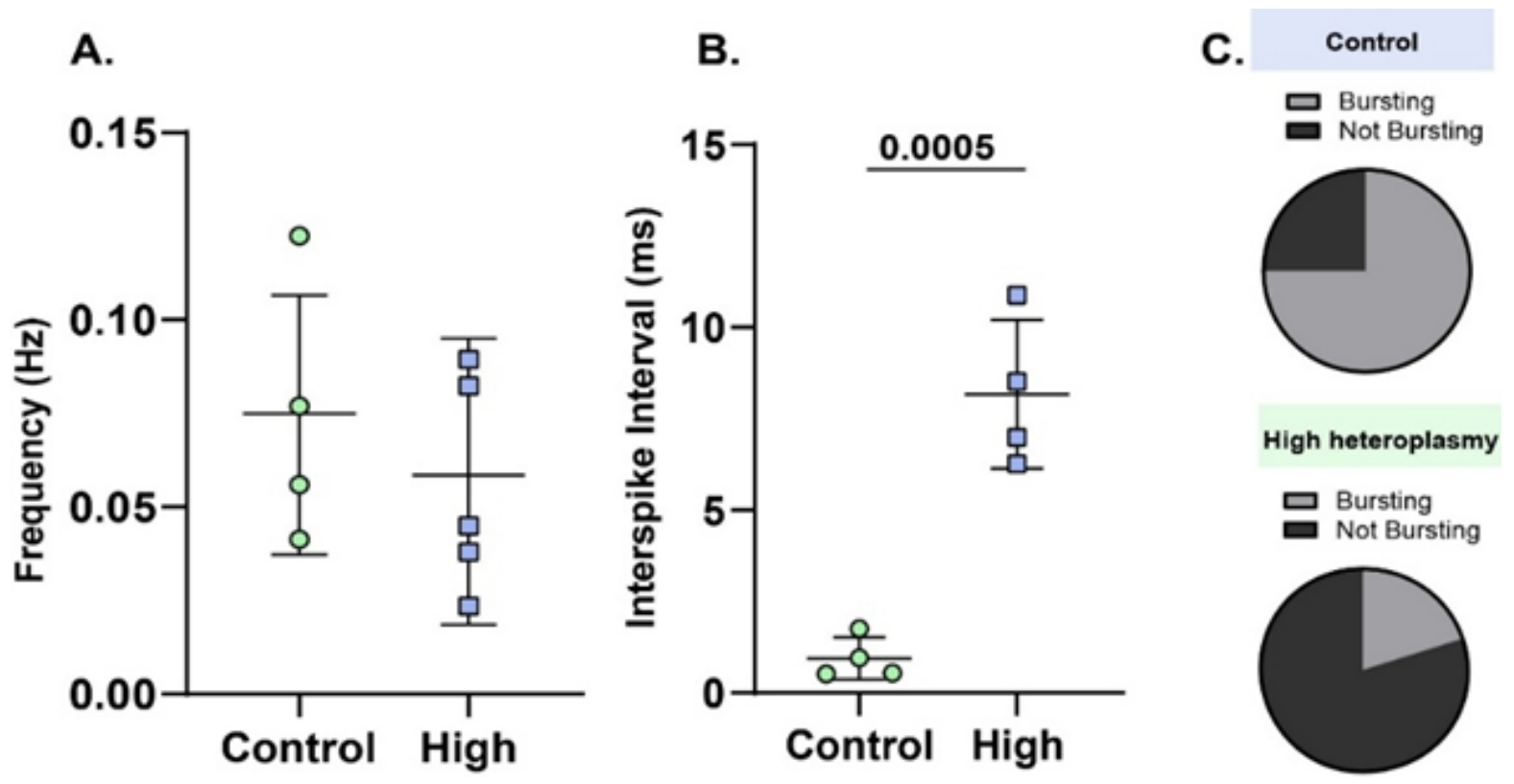


**MEA-based assessment of neuronal activity in control and high-heteroplasmy cultures.** MEA recordings from 200DIV organoids revealed that bursting activity in high-heteroplasmy cultures appeared markedly reduced, presenting as slow, sparse, and disjointed spike patterns. **A.** Mean firing rate (Hz) quantified from MEA recordings in control and high-heteroplasmy organoids. **B.** Interspike interval (ISI, ms) quantified from MEA recordings in control and high-heteroplasmy cultures. Data represent mean ± SEM. **C** Distribution of bursting behaviour detected by MEA. Pie charts show the proportion of electrodes exhibiting bursting activity versus those without detectable bursts in control and high-heteroplasmy cultures.

Regarding the metabolic trajectory, we appreciate the reviewer's suggestion to perform Seahorse assays at earlier developmental stages. We agree that a longitudinal analysis including interim organoid time points and, where feasible, cell type specific measurements would more directly test the proposed transition from early metabolic compensation to later decline and constitutes valuable direction for future work.

4. The inclusion of post-mortem MELAS brain tissue adds translational value by recapitulating the selective loss of deep-layer neurons seen in organoids. However, this comparison is based on a single patient, and as far as I can see, a single tissue section with limited detail on how neuronal loss was properly quantified. Without staining for CTIP2, proper quantification of neuronal loss, and with no specific heteroplasmy percentage reported for the brain tissue, these data remain suggestive but not conclusive.

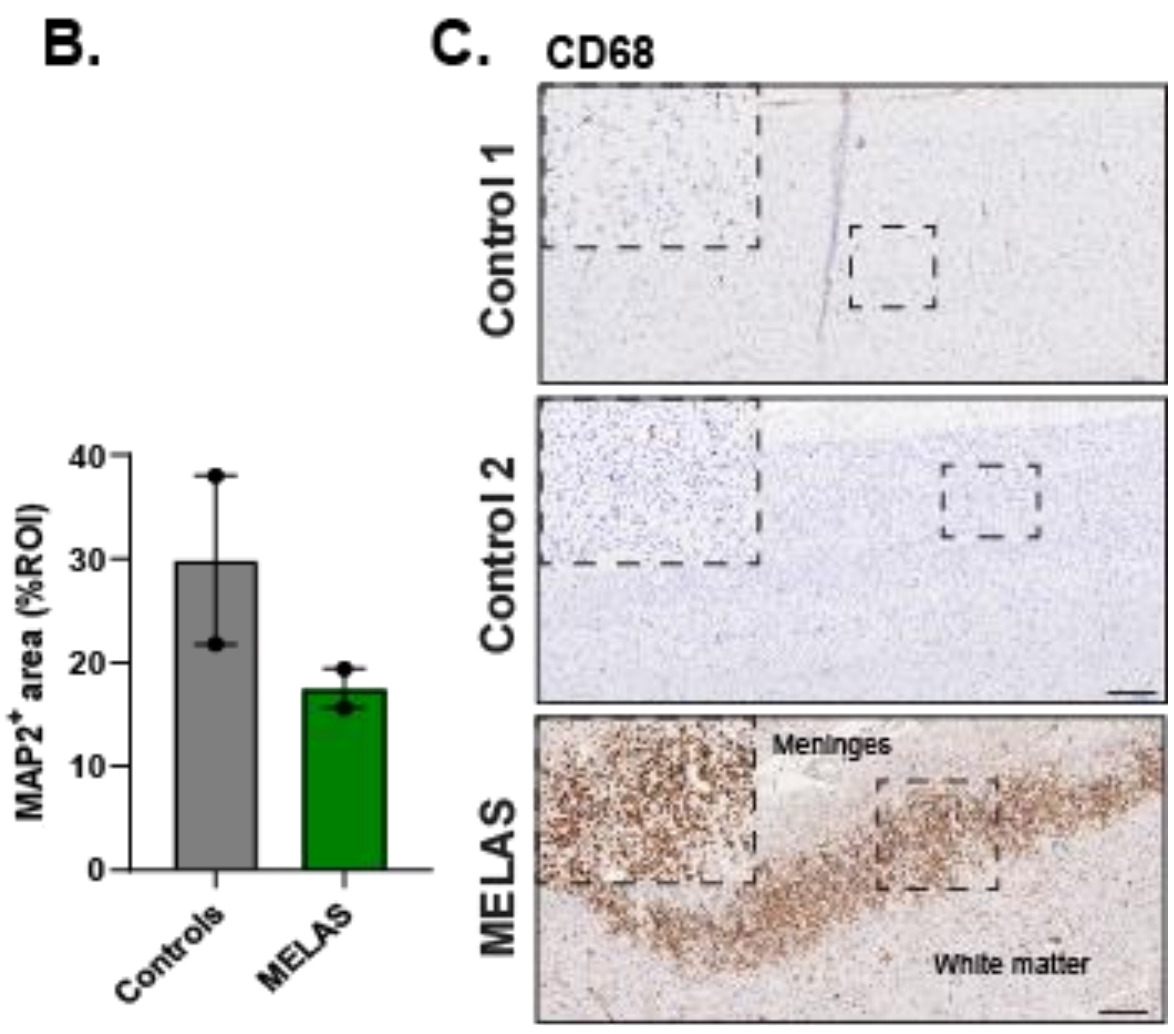


We measured mtDNA heteroplasmy directly in the brain tissue of the patient shown in Figure 5A using FFPE cortical sections and detected a heteroplasmy level of 82%. We attempted to assess CTIP2 and SATB2 in post-mortem FFPE control and MELAS patient brain tissue using both immunofluorescence and immunohistochemistry, testing multiple antigen retrieval conditions (citrate and Tris based), however, reliable staining could not be achieved. To provide additional confirmation of similarities between patient autopsy and organoids, we added the following new data to the manuscript to the Results (page 11) and to the new Figure 5 (B, C).

'Consistent with this, MAP2 positive areas were reduced in MELAS cortex compared to controls (22-38% in controls versus 15-19% in MELAS, Figure 5B). In addition, the presence of CD68 immunoreactivity revealed increased microglial activation in deep cortical layers of the MELAS tissue (Figure 5C). Although this observation is based on a single post-mortem sample, it is broadly consistent with the selective vulnerability of deep layer neurons observed in MELAS organoids (Figure 2D, 2E), suggesting a possible shared mitochondrial mechanism.'

Specific comments and suggestions:

5. Figure 2: The immunofluorescence panels lack logical ordering by heteroplasmy level. A consistent layout across both patient lines would improve clarity.

We have corrected the labels and panel layout in Figure 2B and 2C to improve clarity. Panels are now systematically arranged by patient number (P1 and P2), and, for each individual patient, from the lowest to the highest heteroplasmy.

6. Also, SATB2 appears visually reduced at high heteroplasmy in Fig. 2D, but this is not reflected in the quantifications. it would be helpful if the authors could provide representative examples of their image preprocessing. Given the central importance of these findings, showing what signal is retained or excluded during quantification would strengthen confidence in the data.

We thank the reviewer for raising this point. We would like to clarify that no background subtraction, contrast enhancement, or signal manipulation was applied to the SATB2 channel prior to quantification. As described in the Methods section (Image acquisition and processing), the only correction performed was an illumination adjustment to the DAPI channel to improve nuclear segmentation. All SATB2 measurements were derived from the raw pixel intensities exported directly from the Opera Phenix system. Nuclei were segmented using a pre-trained StarDist model. SATB2-positive nuclei were then classified based on nuclear fluorescence intensity using a threshold derived from inspection of the intensity distribution across the dataset. The threshold was chosen to conservatively separate background and low-level signal from clearly positive nuclei and was applied uniformly to all images. We temporarily adjusted the display contrast and the channel min/max visualization settings in QuPath to better inspect the SATB2 signal and ensure that thresholded pixels reflected true staining rather than background. These adjustments only influence how the image is rendered on-screen and they do not modify the underlying pixel values, segmentation, or subsequent intensity measurements. For figure presentation only, we applied uniform post quantification contrast/brightness adjustments in Affinity Photo to enhance visibility of the SATB2 signal. These modifications were used solely for display purposes and did not influence the quantified results. To increase transparency, we now provide two representative examples for one control and one high heteroplasmy of: (i) the corresponding nuclear segmentation masks used for quantification, (ii) raw SATB2 images (iii) and positive cells attribute to SATB2 after thresholding. This data is included below only for the Reviewer.

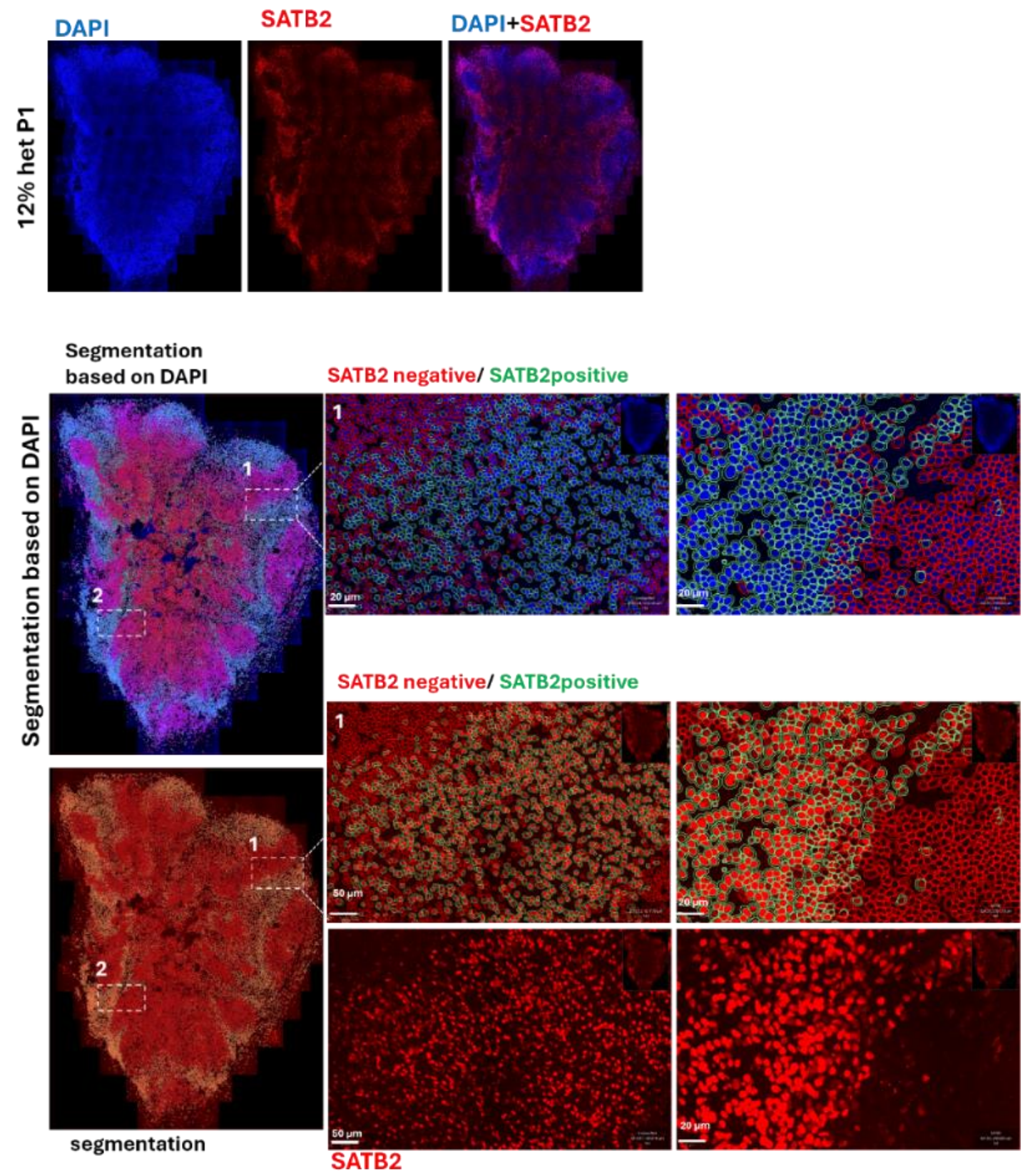
DAPI
SATB2
DAPI+SATB2
12% het P1
Segmentation based on DAPI
SATB2 negative/ SATB2positive
1
2
Segmentation based on DAPI
SATB2 negative/ SATB2positive
1
2
segmentation
SATB2

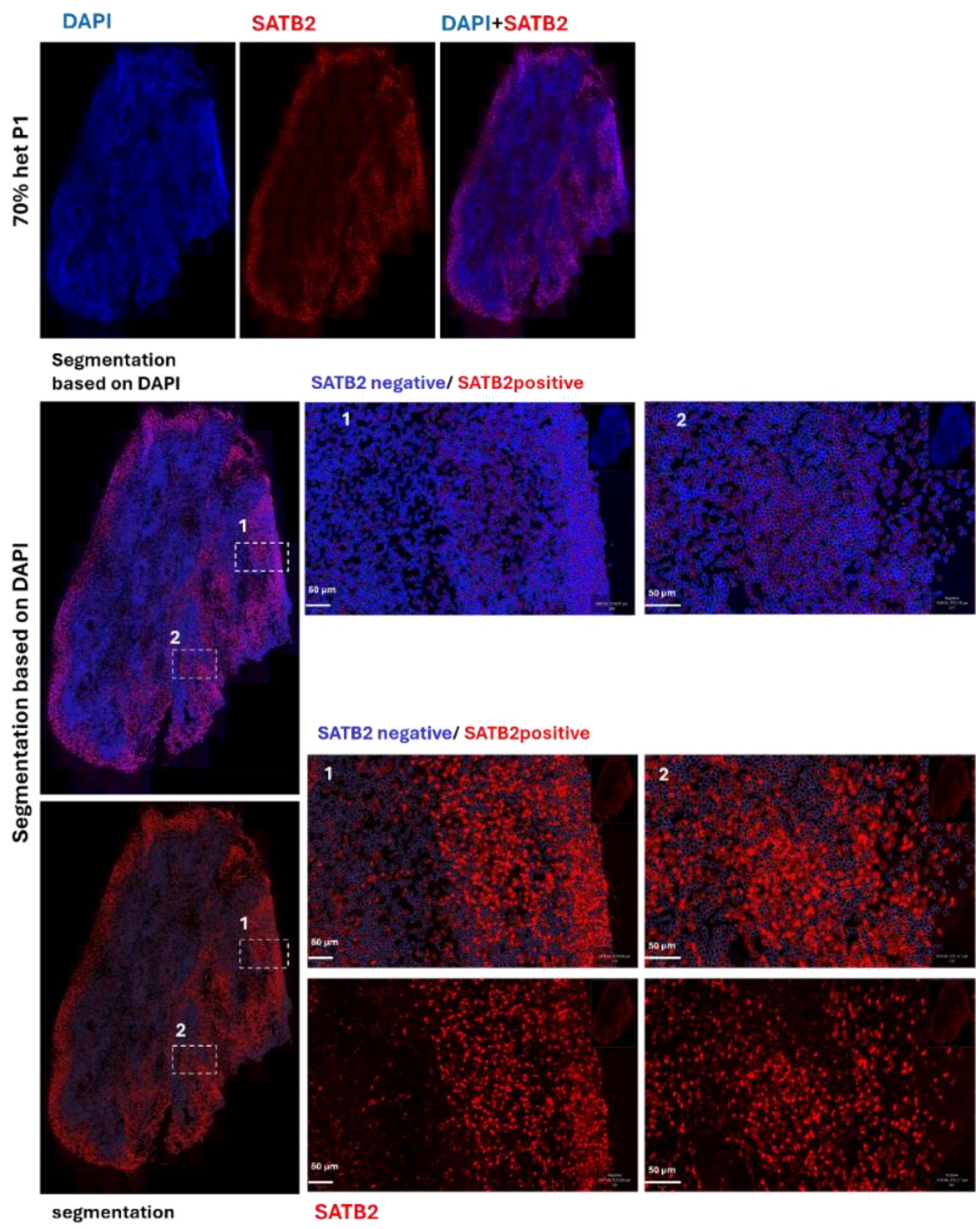
DAPI
SATB2
DAPI+SATB2
70% het P1
Segmentation based on DAPI
SATB2 negative/ SATB2positive
1
2
Segmentation based on DAPI
SATB2 negative/ SATB2positive
1
2
segmentation
SATB2

7. Figure 3 (Seahorse assay): To support the claim of a metabolic shift toward glycolysis, ATP production rates should ideally be calculated (see https://doi.org/10.15252/embr.202256380 or https://doi.org/10.1074/jbc.M116.774471). As ECAR reflects both glycolytic acidification from lactate and CO2 hydration coming from the TCA, a lower OCR/ECAR ratio alone is not sufficient to infer a glycolytic shift. The observed reduction in respiration in high-heteroplasmy organoids may contribute to this ratio, suggesting a potential mixed effect rather than a pure increase in glycolytic flux.

Following the Reviewer's suggestion, we reanalysed our Seahorse data to estimate ATP production rates from oxidative phosphorylation and glycolysis using the Agilent proton efflux framework combined with the approaches described by Mookerjee et al. (JBC, 2017) and Desousa et al. (EMBO Rep, 2023). ECAR values were first converted to proton efflux rates using the Agilent buffer factor, after which glycolytic acidification was calculated based on the established lactate:$H^+$ stoichiometry. Respiratory acidification was corrected using the $CO_2$-derived $H^+$:$O_2$ relationship, and mitochondrial ATP production was derived using the recommended P/$O_2$ value of 5.45.

This analysis confirmed that only high heteroplasmy 100DIV organoids exhibit a significant reduction in mitochondrial ATP production, while glycolytic ATP production remained unchanged relative to controls. As a result, the proportion of ATP derived from glycolysis increased markedly in high heteroplasmy neurons ($ATP_{OXPHOS}/ATP_{glycolysis}$ ratio), indicating a shift in energetic reliance toward glycolysis, even in the absence of a large increase in glycolytic flux.

We have now included this data in the main manuscript in the new Figure 3 C and D (see below new data) and added corrections to the results section (page 7-8) to better fit the new calculated ATP rates:

'Real time metabolism was monitored by the Seahorse assay, which was performed on cells dissociated from organoid slices. Using the OCR and ECR traces we calculated ATP production rates as described previously[23,24]. In both patients, total ATP production increased in medium heteroplasmy but remained unchanged in high heteroplasmy organoids at 100DIV compared to controls. When separating ATP sources, glycolytic ATP production was elevated in medium heteroplasmy organoids, while mitochondrial ATP production was specifically reduced only in high heteroplasmy. As a consequence the $ATP_{OXPHOS}/ATP_{glycolysis}$ ratio decreases with increasing heteroplasmy, indicating a shift in glycolytic reliance (Figure 3C and D). '

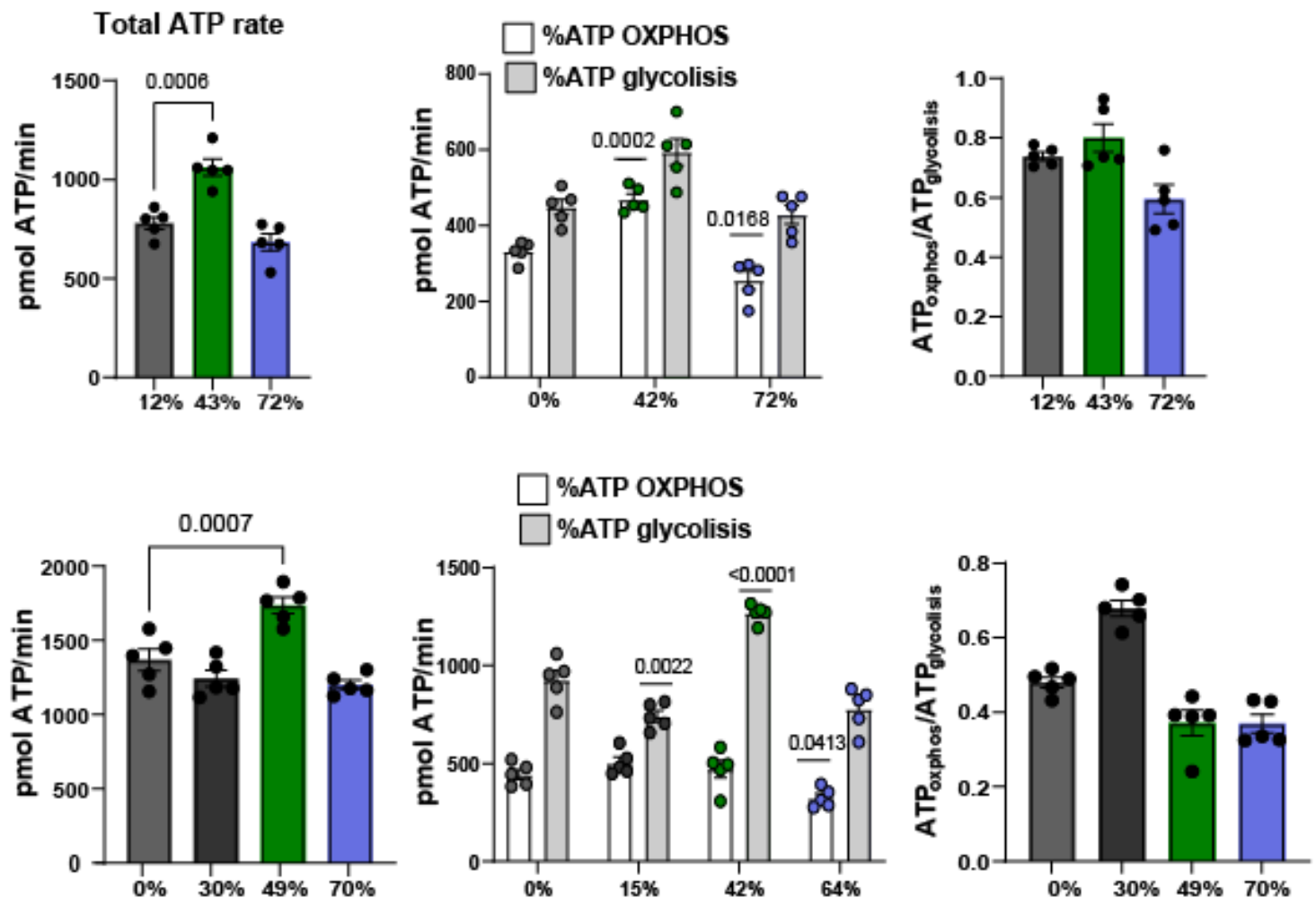


8. Is there a reason why the authors have only looked at complexes I, II, IV, and V, but ignored complex III in their profiling? (see figures 3 and 6)

We have now performed Western blotting on 100DIV organoids and included representative blots and quantification in new Figure 3 A and Figure S3 A. These analyses demonstrate that Complex III levels do not show a statistically significant difference amongst different heteroplasmy organoids.

We have included the following description at page 7:

'In contrast, SDHB (Complexes II), UQCRC1 (Complex III), ATP5A and MTATP6 (Complex V) remained unchanged in all organoids (Figure 3 A and Figure S3 BC). These results confirm that the extent of OXPHOS disruption depends on heteroplasmy levels, with Complex I and IV being more susceptible to elevated mutation burden.'

9. I appreciate that the authors quantified mtDNA copy numbers in neurons and astrocytes, though it's a bit unclear why astrocytes were the only glial population included. Expanding this analysis to other glial cell types in future studies would be great, as glial cells are often underappreciated. I look forward to more cell-type–specific insights in this area.

We agree with the reviewer and fully acknowledge the importance of glial diversity in mitochondrial research. As the central aim of our study was to investigate neuronal vulnerability across heteroplasmy ranges, our analyses were primarily focused on neuronal populations. Astroglial cells were included as an additional example of a post mitotic glial cell

type to provide a contrasting reference point. At the developmental stage examined in this study (100DIV), our cortical organoids predominantly contain neurons and a small population of astrocytes and glial progenitors. Oligodendrocyte progenitor cells and oligodendrocytes arise at later stages of organoid development, and as such, they could not be properly assessed within the current experimental framework. Mesodermic components, such as microglial cells, have been suppressed in this organoid culture protocol, in order to maintain the consistency and reproducibility of neuroectodermic components across organoids, for high fidelity comparisons. Examining mtDNA regulation and mitochondrial function across a broader range of glial subtypes represents an important but more time-consuming direction for future, which we would like to pursue in a separate study.

10. Line 303: The statement that "these findings well reflect the loss of $CTIP2^{+}$ deep layer neurons found in organoids" is misleading; CTIP2 staining was not performed in the post-mortem tissue, and the loss of DLNs was not quantified. With an n of 1, this could reflect tissue quality rather than biology.

We agree with the reviewer; however we had technical difficulties with CTIP2 immunostaining in the post-mortem tissue and we cannot directly assess or quantify $CTIP2^{+}$ DLN in the MELAS cortex. We therefore agree that the original statement suggesting a specifical loss of $CTIP2^{+}$ deep-layer neurons in post-mortem tissue was overstated. We have revised the manuscript to remove this wording and to more carefully reflect the available evidence. The post-mortem data are now described as showing reduced MAP2 immunoreactivity in regions corresponding to dense nuclear layers (new Figure 5A, white arrows), together with a quantitative reduction in total MAP2-positive area in high-heteroplasmy tissue, indicating reduced neuronal process integrity in these regions rather than definitive neuronal loss. Unfortunately, no additional m.3243A>G post-mortem brain tissue was available for validation. We added it to the Results on page 11 (please see in our response to point 4.)

11. Figure S5B (GFAP staining): The image is difficult to interpret without a control comparison.

We thank the reviewer for this valuable suggestion. We have now re-imaged the GFAP staining in the MELAS case and added GFAP stained sections from two neurologically normal control brains (a 57-year-old male and an 85-year-old male, both without reported brain pathology) to

allow direct comparison. As shown in the revised Figure S5 B, the MELAS cortex displays a clear increase in GFAP positive astrocytes and more prominent astrocytic processes compared to both controls, consistent with astroglial responses in the patient tissue. We have updated the figure and legend accordingly.

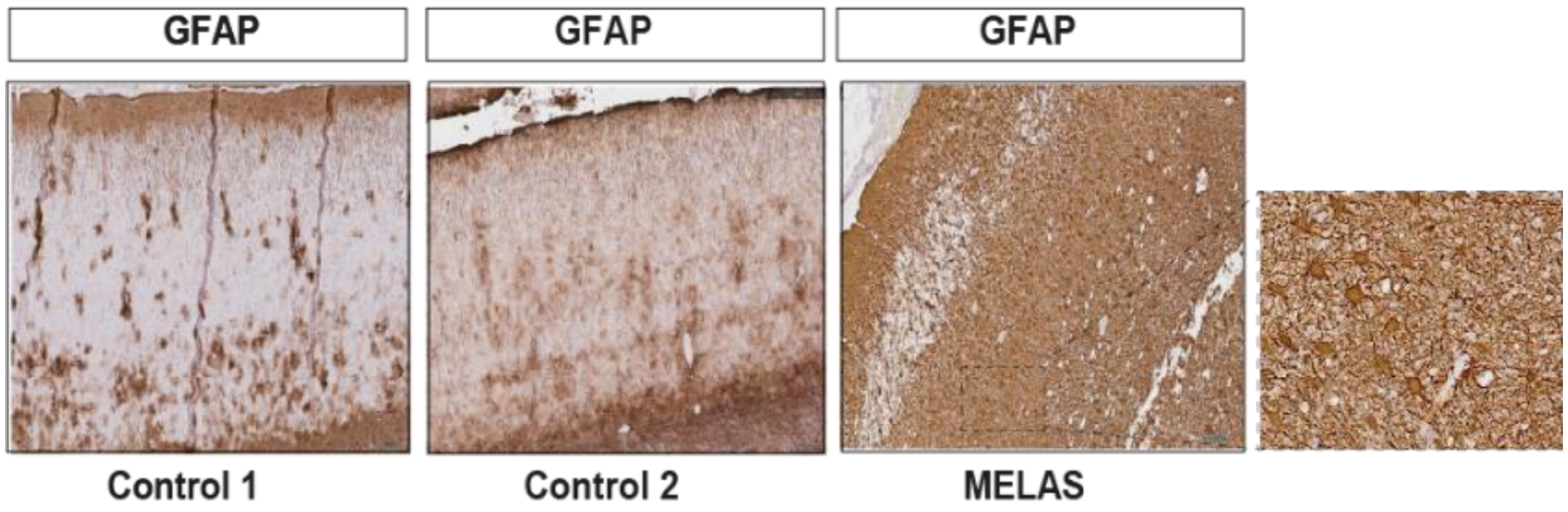


12. Figure 6 F-H: Visual inspection suggests the data are not normally distributed. The authors report using non-parametric tests when assumptions are not met (line 873), but Anova is still used in this figure. Please clarify whether assumptions (normality, equal variance) were tested (e.g., via Q-Q plots or residual analysis), and consider using a non-parametric alternative if necessary.

We assessed data distribution using D'Agostino-Pearson and Shapiro-Wilk normality tests in GraphPad Prism, which indicated significant deviation from a normal distribution; inspection of Q-Q plots was consistent with this. Based on this we used a non-parametric approach for the statistical comparison in Figure 6F-H (now Figure 6 G-I). Group differences were assessed using unpaired, two-tailed Mann-Whitney test. The reference to Anova in the figure legend was an error which has been corrected in the revised manuscript.

13. Seahorse methods: FCCP concentration is not reported. Maximal respiration/spare capacity cannot be interpreted without this information, and FCCP dose should ideally be titrated. Also, how was total DNA quantified with Hoechst, was it pixel-based or a nuclei count? Also, what do the authors mean with "ATP production"? Is it ATP-linked respiration, i.e. baseline - post-oligo? Again, calculating ATP production rates for OxPhos and glycolysis would help with the interpretation.

We thank the reviewer for these helpful comments. We have included the FCCP concentration used in the Seahorse Mito Stress Test (1 µM) in the Methods section. This concentration is widely used for neuronal cultures and provides reliable uncoupling without inducing toxicity.

Regarding normalization, we used a pixel-based quantification of Hoechst fluorescence to estimate total DNA content per well (included in the methods section). We initially attempted BCA protein quantification however, Matrigel coating introduces substantial protein background that interfered with accurate measurements, leading us to adopt Hoechst based normalization. Pixel-based Hoechst quantification is frequently used for Seahorse assays in neuronal and organoid derived cultures because it enables accurate normalization of total DNA without interference from extracellular matrix components.

"ATP production," refers to ATP-linked respiration, calculated as the difference between basal OCR and OCR following oligomycin treatment in the Mito Stress Test. As suggested previously by the reviewer we have calculated ATP rates which are now included in the new Figure 3 C and D.

Minor corrections:

We thank the reviewer for pointing out these small errors. We addressed all these minor issues in the manuscript.

14. Fig1C batches 1 and 3 have the same color-code

15. Panel Fig 2G has plus-symbols, probably from text formatting

16. The legend in Fig 2H is cut off

17. Fig. S3C where is the 69% heteroplasmy coming from? --> please check for consistency throughout the manuscript

18. Figure 3E/F: The legend is missing detail about the color code. It's unclear what the green and blue represent, especially as both colors appear within each patient group.

19. Figure S4B (line 298): Should be labeled as Figure S5B. Please double-check figure references throughout.

**Reviewer #3 (Remarks to the Author):**

This study aims to establish and characterise a cellular model of heteroplasmic mutation of the mitochondrial MT-TL1 gene (m.3243A>G) using human patient iPSC-derived cerebral organoids. Through single-cell transcriptome analyses and comparisons of different heteroplasmy levels in these iPSC-derived cerebral organoids, the authors concluded that the m.3243A>G variant does not lead to transcriptional changes associated with cell differentiation, except for a reduction in the proportions of DL cells. Immunostaining analyses confirmed the reduction of CTIP2+, DL-identity marker, cells in organoids with high levels of heteroplasmy. Additionally, the authors found that reduced mitochondrial OXPHOS proteins and reduced mitochondrial oxygen consumption rate were associated with a high level of heteroplasmy. Furthermore, the author observed an increased number of TUNEL-positive cells in CTIP2+ cells, which may explain the reduced proportion of DL neurons in organoids with high heteroplasmy, along with axonal structural abnormalities. The authors addressed important and timely questions to gain a deeper understanding of the pathogenesis associated with MT-TL1 gene mutations. Their findings have the potential to enhance our understanding of how mitochondrial heteroplasmy impacts cellular function. However, to support the conclusion presented in the manuscript, the data in several sections (as detailed below) would need substantial strengthening, likely involving new experiments.

We thank the reviewer for the positive comments.

Major points:

1. Cell-type specific vulnerability:

The author demonstrated a reduction in deep layer (DL) and CTIP2+ neurons in high heteroplasmy organoids through transcriptome analysis (Figure 1E) and immunostaining (Figures 2B-E). Based on the increased proportion of TUNEL+ cells among CTIP2+ neurons (Figures 5B,C), the author concludes that high heteroplasmy promotes apoptosis in deep layer neurons, leading to a specific loss of DL neurons in these organoids.

a) To strengthen the author's conclusion and better interpret the results of Figure 2E, it would be beneficial to compare neuronal populations across organoids with varying levels of heteroplasmy. This comparison could involve immunostaining for a pan-neuronal marker, such as NeuroD2.

We agree with the reviewer that it is important to distinguish between a global reduction in neurons and a selective loss of $CTIP2^+$ DLN. We have assessed neuronal populations using a pan neuronal marker, TUJ1 (βIII-tubulin) in Western blotting (Figure 1F and G), which reflects overall neuronal content in 100DIV organoids, and shows no significant difference in TUJ1 levels across heteroplasmy conditions.

In addition, we have performed NeuN immunostaining in 100DIV organoids which shows no significant change in the proportion of $NeuN^+$ cells across different heteroplasmic organoids (Data also included below). We have added this analysis in the manuscript to Figure 1 F, G and new Figure S2E, F. These data indicate that overall neuron abundances are similar across different heteroplasmy levels, whereas $CTIP2^+$ deep layer neurons are selectively reduced only in high-heteroplasmy organoids.

The text in the results section has been revised accordingly (page 6) 'Together these findings indicate that high m.3243A>G heteroplasmy preferentially affects deep-layer neuronal populations, while overall neuronal abundance, asset using the pan-neuronal marker TUJ and the neuronal nuclear marker NeuN, remains largely preserved across heteroplasmy levels (Figure 1F, G and Figure S2E, F).'

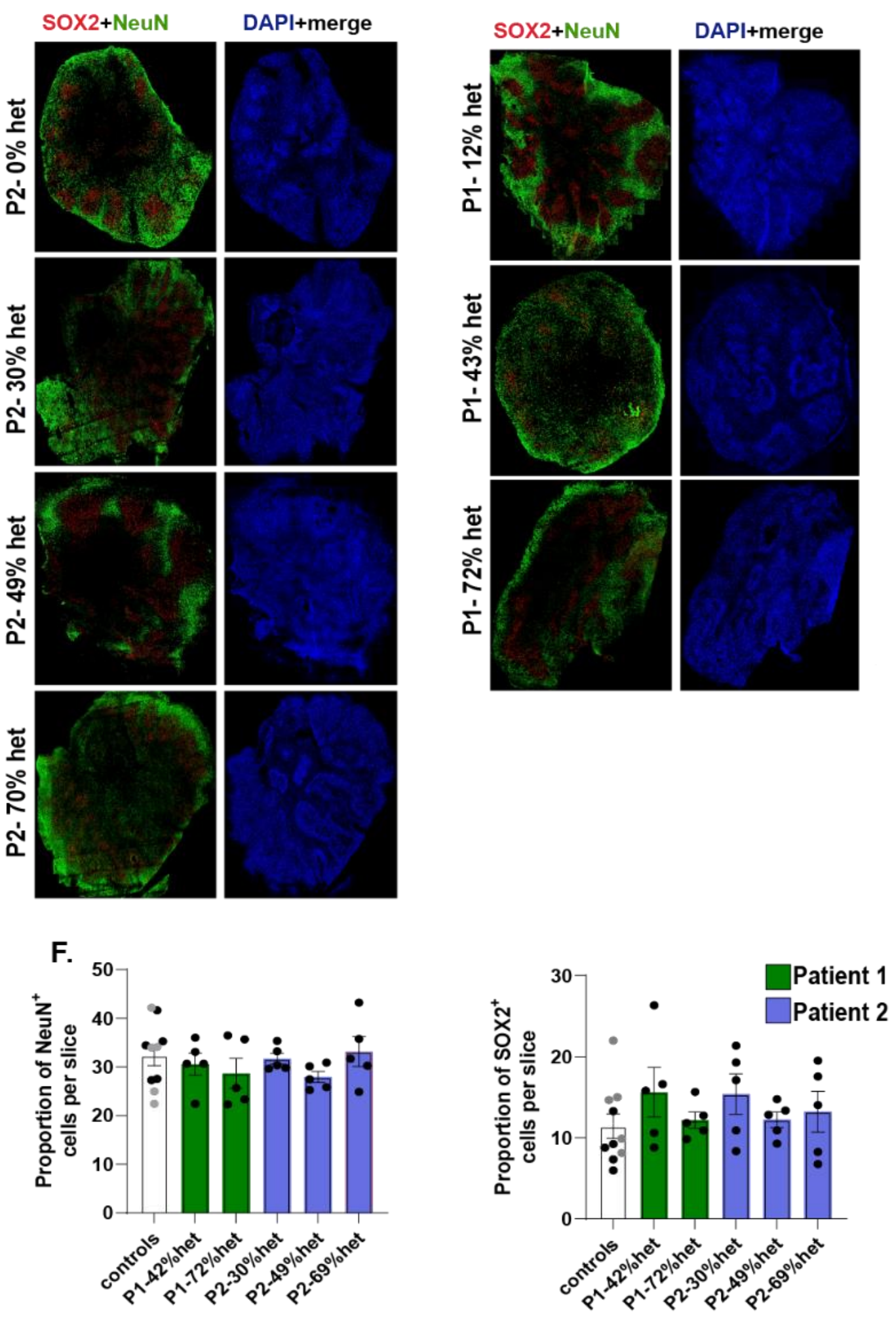


b) Furthermore, what is the impact of high heteroplasmy on the proportion of TUNEL+ cells in other cell types, such as upper layer (UL) neurons and glia? It is essential to determine whether the effects are specific to DL neurons.

We agree with the reviewer that it is important to determine whether the increase in apoptosis is specific to DL neurons or also affects other neuronal types. To address this, we performed TUNEL co-immunostaining with SATB2 to assess apoptosis in UL neurons. Quantification showed no significant increase in the proportion of SATB2$^{+}$/TUNEL$^{+}$ cells in high heteroplasmy organoids compared to controls (please see new data below). This supports the conclusion that high heteroplasmy preferentially affects DL neurons rather than UL populations.

We have included this data in Figure S5 C and D and at page 11:

'In contrast quantification of SATB2$^{+}$/TUNEL$^{+}$ proportion of cells shows no change in high heteroplasmy organoids (Figure S5C and D).'

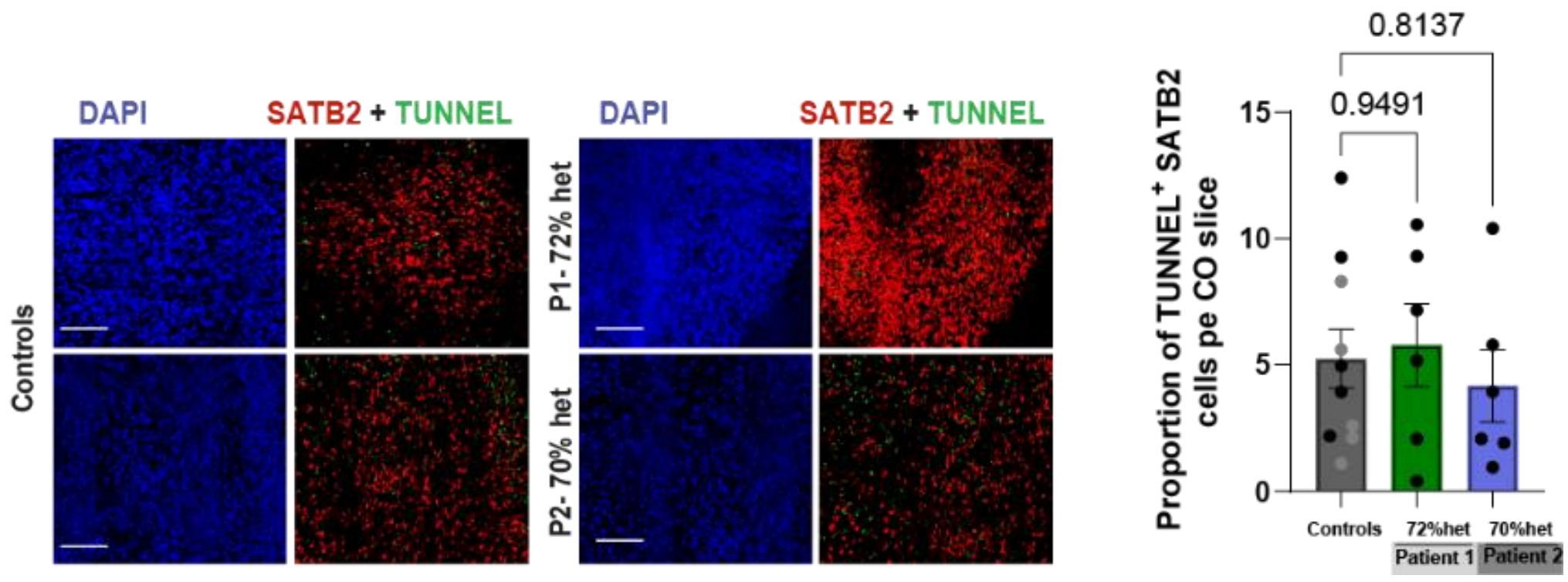


c) Additionally, another possibility, such as the reduction in the production of DL neurons caused by the m.3243 A>G mutation, has not been explored.

We thank the reviewer for this suggestion. To test whether the reduction in DL neurons could reflect impaired development rather than apoptosis, we analysed DL and UL neuron numbers at an earlier stage, prior to the time point at which we observed apoptosis of DLN. Specifically, we performed CTIP2 and SATB2 immunostaining at DIV70 and quantified the proportion of CTIP2$^+$ DL and SATB2$^+$ UL neurons. We found no significant differences between control and high-heteroplasmy organoids at this stage (new Figure S5E and F), arguing against a primary defect in deep-layer neuron generation. This is consistent with our scRNA-seq analysis, which shows preserved specification and maturation trajectories from radial glia to DL and UL neuronal states across heteroplasmy levels, with the expected post-mitotic neuronal signatures (Figure 2F, H; Figure S2G, H).

Together, these data support that high m.3243A>G heteroplasmy that DL neurons reduction is because of selective later stage vulnerability and loss of CTIP2$^+$ neurons. We have added the new DIV70 CTIP2/SATB2 data and clarified this point in the Results section (page 11).

'Consistent with intact generation of cortical projection neuron subtypes, CTIP2 and SATB2 immunostaining at 70 DIV showed comparable proportions of DLN and ULN neurons in control and high heteroplasmy organoids (Figure S5E and F). Thus, our data support a selective late-stage loss of CTIP2$^+$ DLN neurons under high m.3243A>G heteroplasmy.'

2. a) Links between levels of heteroplasmy m.3243 A>G and mitochondrial defects: Several reports indicate differences in mitochondrial activity and metabolism between cortical progenitors and neurons, with neurons exhibiting higher oxidative phosphorylation (OXPHOS)

activity than their progenitors. To understand the impact of high heteroplasmy on neuronal mitochondrial functions, it is important to include information about the ratio of neurons to progenitors in the experiments shown in Figure 3 (WB and Seahorse assays).

We thank the reviewer for raising this point. To determine whether differences in neuronal and progenitor composition could influence the mitochondrial phenotypes seen in Figure 3, we have quantified the number of NeuN$^+$ mature neurons and SOX2$^+$ progenitor cells in 100DIV organoids. NeuN$^+$ and SOX2$^+$ immunostaining showed comparable proportions of neurons and progenitor cells across all conditions. These data are now included in the new Figure S2E and F. Together these findings indicate that neuron to progenitor ratio is largely preserved across heteroplasmy levels at 100DIV. We therefore suggest that the mitochondrial proteins level alterations and the detected bioenergetic defects reflect intrinsic mitochondrial alterations rather than differences in cellular composition. This has been clarified in the results section at page 8:

'Because the proportions of mature neurons (NeuN$^+$) and progenitor cells (SOX2$^+$) were comparable across heteroplasmy levels in organoids at 100DIV (Figure S2E,F), the OXPHOS and ATP defects observed in high-heteroplasmy organoids are most consistent with intrinsic mitochondrial dysfunction rather than differences in cellular composition.'

b) Additionally, conducting the Seahorse assay with isolated neurons to measure oxidative activity could further strengthen the authors' conclusions. In this line, the authors highlighted the differences in mtDNA copy number between whole organoid-derived cells and isolated neurons in Figures 3E and 3F.

We agree that Seahorse measurements in isolated neurons would be informative for assessing neuron-intrinsic mitochondrial defects. However, isolation of neurons from organoids yields highly variable cell numbers and attachment efficiencies, which limits the robustness and reproducibility of Seahorse measurements in this setting. To address this point, we therefore performed Seahorse assays on 2D derived CTIP2$^+$ neurons generated from the same patient iPSC lines. This data is now included in the new Figure 6D, E and Figure S6A-D and included

In contrast to cortical organoids, DLN neurons showed a clearly more OXPHOS dependent metabolic profile, with the majority of ATP derived from mitochondrial respiration. Total ATP

production was reduced in high-heteroplasmy neurons, driven by a decline in mitochondrial ATP generation. Glycolytic ATP production did not increase to compensate for this loss.

We added this in the results section at page 12: 'Seahorse analysis demonstrated a heteroplasmy-dependent reduction of total ATP production. (Figure 6D, E, Figure S6 A-D). Although OXPHOS remains the primary source of ATP, its contribution was significantly decrease in high heteroplasmy neurons. The absence of a compensatory increase in glycolytic ATP indicates a failure of metabolic reprogramming and highlights an intrinsic vulnerability of these neurons to mitochondrial dysfunction (Figure 6D, E).'

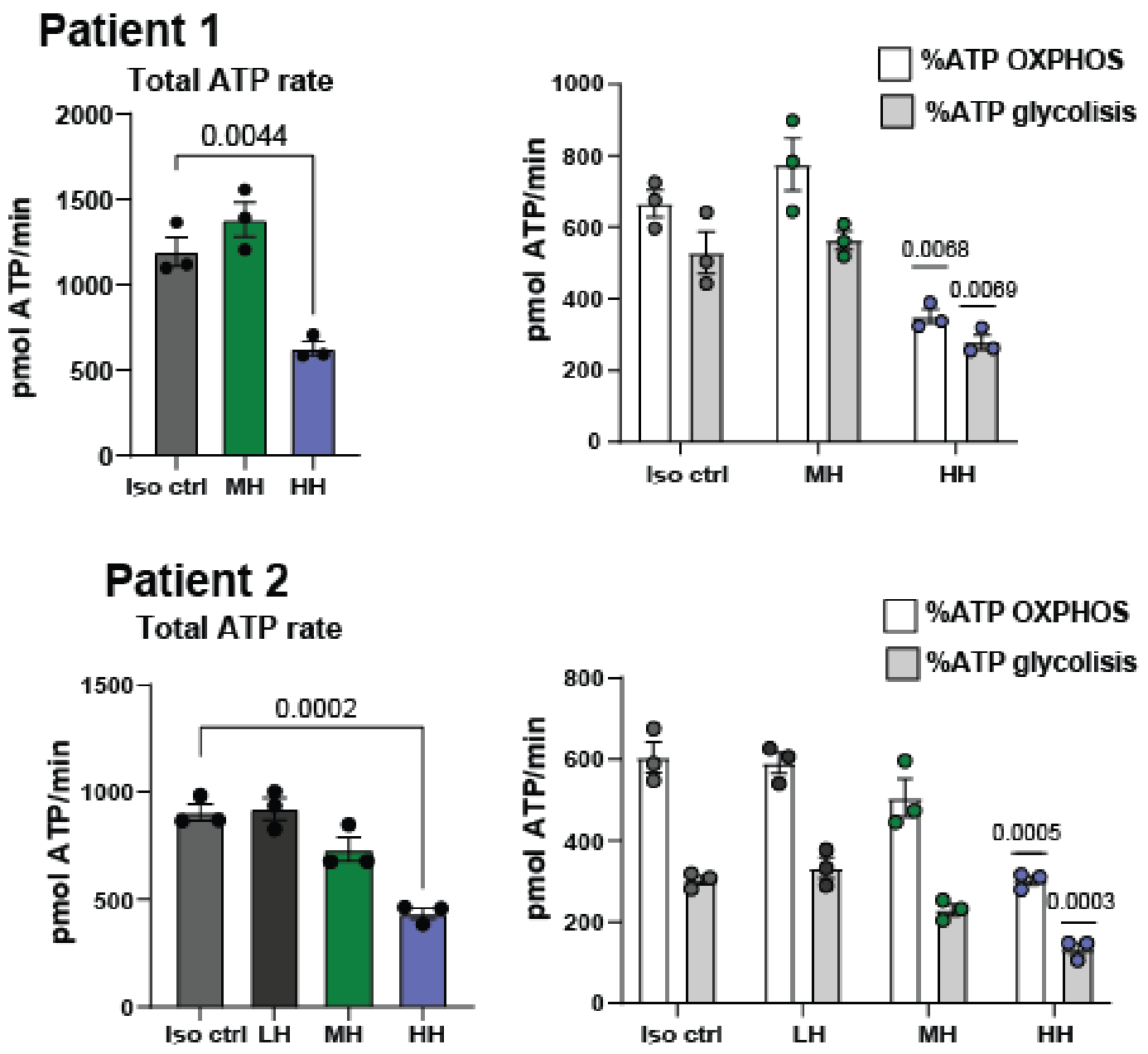


c) Furthermore, analysing the expression levels of other proteins coded by mitochondrial DNA would help generalise the effects of high levels of heteroplasmy of m.3243 A>G mutation.

In addition to MTCO1, we have now examined several further mtDNA-encoded OXPHOS subunits by Western blot. For Complex IV, MTCO2 was significantly reduced in high heteroplasmy organoids, mirroring MTCO1 (Figure 3A, B and Figure S3B, C). For complex V, MTATP6 was significantly decrease in high heteroplasmy organoids from patient 2. These additional mtDNA encoded subunits further support a generalized impairment of mitochondrial encoded OXPHOS components at high heteroplasmy. This data was added in the new Figure 3S B and C. We have also attempted to address the Complex 1 subunits ND1 and ND6, however available antibodies produced non-specific bands and very variable patterns of bands across repeats consistent with the technical challenges associated with detecting these highly hydrophobic proteins via Western Blotting (see below). We therefore focused our Complex I

analysis on NDUFB8 (Figure 3A, B), a frequently used nuclear encoded subunit that provides a robust and widely used proxy for Complex I integrity.

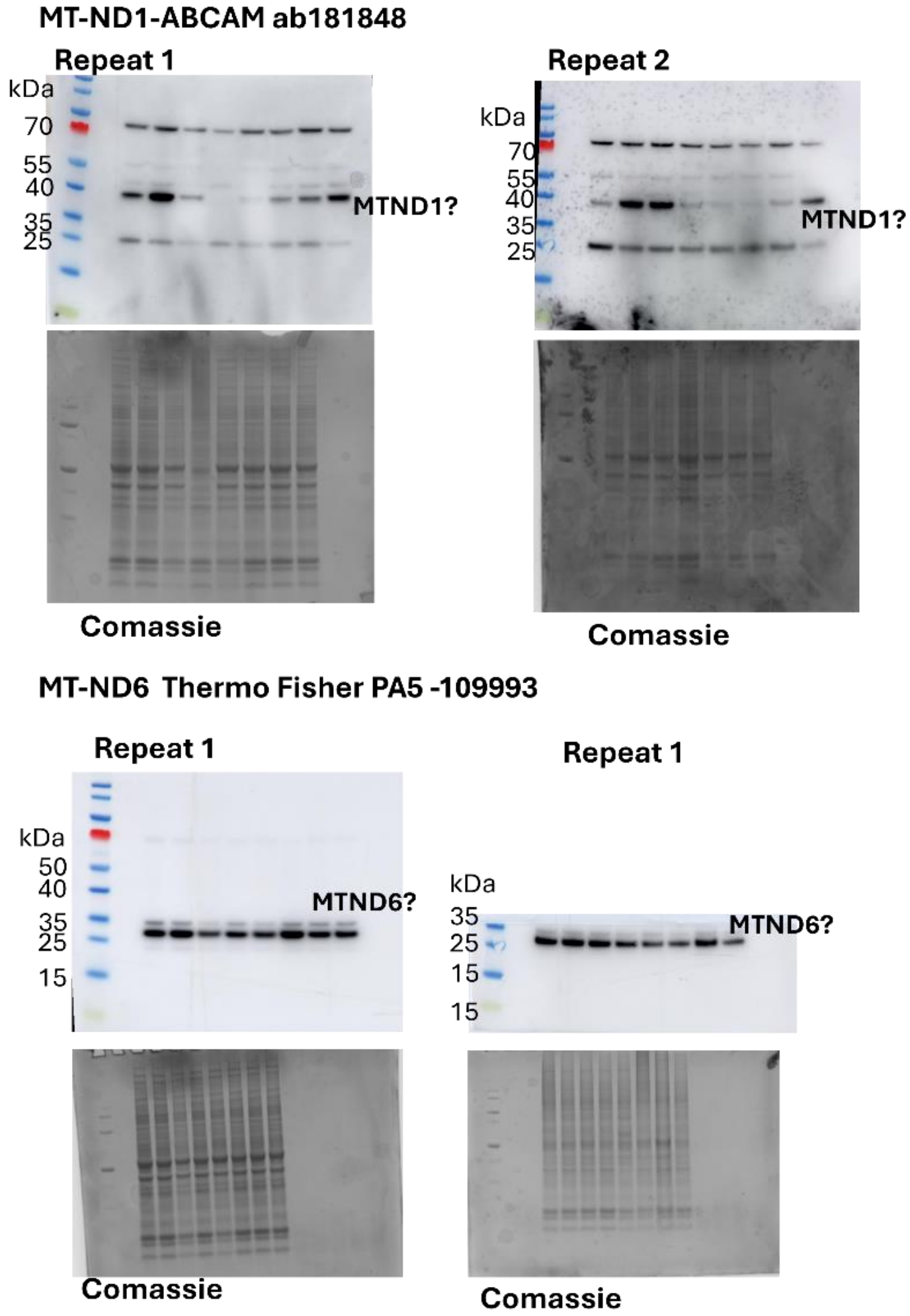


3. MELAS patient phenotype:

Identifying the conserved features between the organoid model and MELAS patients is crucial for accurately replicating the pathology in the organoid model. In this context, evaluating the proportion of SATB2+ and CTIP2+ neurons, as well as the presence of axonal abnormalities in MELAS patient tissue is essential.

We agree with the reviewer that these analyses would be relevant to the MELAS patient autopsy samples. To this end, we attempted to assess layer specific neuronal markers in FFPE control and patient tissue using both immunofluorescence and immunohistochemistry, testing multiple antigen retrieval conditions (citrate and Tris based); however, we were technically unable to obtain a detectable staining at any conditions.

Minor points:

We thank the reviewer and we corrected these minor issues.

4. Why do the "S7 DEGS DLN5B vs ULN high h" contain the same genes, such as CDH7, GRIA2, and DPP10, in both UP-regulated and DOWN-regulated groups? Is this associated with the Figure 4J (right) result? If so, why are MT-DN2, ND3, and ND5 not listed in the S7 table?

Table S7 is associated with figure 4J. The overlap between up- and down-regulated genes in Table S7 resulted from an inadvertent duplication during table compilation. We have now corrected Tables S7 and S8.

5.Typos:

Line 93; Line 226 Figure 3SD; Line 231 Figure 3SE, Line 295 Figure S4A, Line 298 Figure S4B

Dear Editor and Reviewers,

We thank you for the careful and constructive evaluation of our manuscript and for its acceptance.

We have revised the manuscript in line with the reviewers' final comments. Please find our point-by-point responses below.

We also provide the requested brief summary of the main findings for use on the journal website:
"A human brain organoid model of a mitochondrial DNA mutation shows that neuronal vulnerability increases with mutation load, revealing cell type-specific susceptibility underlying neurodegeneration."

Thank you very much.

Sincerely,

Denisa Hathazi, Andras Lakatos and Rita Horvath

Reviewer comments

**Reviewer #2** (Remarks to the Author):

Thank you for the detailed and thoughtful responses to all of my comments. Overall, my concerns have been adequately addressed, and the revisions substantially strengthen the manuscript.

The statement that MT-ATP6 "remained unchanged" across all organoids does not appear accurate based on the quantification, where MT-ATP6 is reduced by ~40% in the high-heteroplasmy condition. I recommend revising this wording (e.g. "moderately reduced" instead of "unchanged") and briefly acknowledging that the concordant reduction across mtDNA-encoded markers (MT-CO1, MT-CO2) could reflect a genome-level effect on mtDNA-encoded protein abundance (independent of copy number), rather than a strictly complex-specific effect.

We thank the reviewer for the comment. We have now acknowledged that MT-ATP6 is moderately reduced in high heteroplasmy organoids. We have also now incorporated a statement acknowledging that the observed reduction across studied mtDNA encoded proteins,

such as MT-CO1, MT-CO2 and MT-ATP6 may reflect a broader effect on mtDNA-encoded protein abundance, rather than a strictly complex-specific vulnerability.

These changes have been made in the revised manuscript (page 7).

**Reviewer #3** (Remarks to the Author):

The authors addressed all reviewers' points, and their results support their conclusion. This work is timely and contributes to the broad field of neurodevelopmental biology.

We thank the reviewer for their positive and encouraging comments.
