# Supplementary material for: Mitochondrial DNA heteroplasmy drives cortical neuronal disturbances in human organoids harbouring the common m.3243A>G mutation
Source: Nat Commun. 2026 Jun 21;17:7840. doi: 10.1038/s41467-026-74103-y (PMC13438594; doi:10.1038/s41467-026-74103-y)
Supplement: Supplementary file 4 [file 41467_2026_74103_MOESM1_ESM.docx]

**Supplementary figures**

**Figure S1. A.** Representative agarose gel for determining the m.3243A>G mutation in iPSCs. Screening of the m.3243A>G mutation was done by PCR‐RFLP where a 528 bp PCR fragment is digested with ApaI. The wild‐type PCR product does not contain ApaI restriction site, whereas PCR product containing the m.3243A>G mutation is cleaved into two fragments of 234 and 294 bp in length; **B.** Phase contrast imaging of whole organoids where expanded neuroepithelia show similar bud-formation between cortical organoids at 10 DIV; **C.** Representative immunofluorescence images of cortical organoids at DIV 30, showing the palisade-like organization of SOX2⁺ progenitor cells (violet), surrounded by immature TUJ⁺ neurons (red) and NESTIN⁺ progenitor cells (green). Scale bar = 50 µm; **D.** Representative immunofluorescence images of ALI-COs at DIV 100, showing TUJ⁺ (green) neurons and GFAP⁺ (red) glia across three biological replicates per group. Scale bar = 200 µm;

**Figure S2**. **A.** Representative immunofluorescence images of cortical plate regions at DIV 75, showing the forebrain identity marker FOXG1 (green) and early-born CTIP2⁺ neurons (violet), with insets highlighting cortical plate organization. Scale bar = 50 µm; **B.** Average gene expression of marker genes for various subpopulations identified in 11 unbiased clusters by scRNA-seq within the merged dataset of control and C9 ALI-COs (n = 119,591 cells). Cell annotation: ULN, upper-layer cortical neurons; DLN5B and DLN5A, deep-layer neurons (layer 5, subtypes A and B); DLN6, deep-layer neurons (layer 6); IN, interneurons; yULN, young upper-layer neurons; IP, intermediate progenitors; RG, radial glia; oRG, outer radial glia; CP, choroid plexus; Ui, unidentified cells; **C.** UMAP representations of scaled expression levels and distribution of key marker genes of major cell-types identified; **D.** Representative immunofluorescence images of laminar organization in cortical organoid slices at DIV 100, where SATB2⁺ cells (red) mark ULNs and CTIP2⁺ cells (green) label DLN5 neurons with scale bar = 200 µm; **E.** Representative immunofluorescence images of ALI-COs at DIV 100, showing NeuN^+^ and SOX2^+^. Scale bar = 400 µm; **F**. Bar plots quantifying NeuN⁺ (left) and SOX2^+^(right) cells, normalized to the total number of cells across the entire ALI-CO cryosection. Data represent the mean ± SEM; *n* = 5 independent ALI-COs per heteroplasmic line and isogenic controls from different batches. Statistical analysis: Two-sided one-way ANOVA followed by Dunnett’s post hoc test (overall p-values indicated in the graphs). **G.** Bar plots indicate proportion of cells mapping to transcriptional profiles of various fetal ages in scMAP; **H.** UMAP representation of inferred developmental trajectories in low, medium and high heteroplasmy obtained by using Monocle3.

**Figure S3. A.** Quantification of WB data for SDHB (Complex II) and UQCRC1 (Complex III) ATP5A (Complex V). Data were normalized to TOM20 to account for mitochondrial content. Data represent the mean ± SEM; *n* = 3 ALI-COs from three independent batches, with two organoids pooled per sample. Statistical analysis: one-way ANOVA followed by Dunnett’s post hoc test (overall ANOVA *p*-values indicated in the graphs); **B**. Representative western blot (WB) images for MT-ATP6, MT-CO2, and TOMM20. Corresponding molecular weights are displayed on the right side of the blot; **C.** Quantification of WB data for MT-ATP6 (Complex V) and MT-CO2 (Complex IV). Data were normalized to TOM20 to account for mitochondrial content. Data represent the mean ± SEM; *n* = 4 ALI-COs from three independent batches, with two organoids pooled per sample. Statistical analysis: one-way ANOVA with followed by Dunnett’s post hoc test (overall ANOVA *p*-values indicated in the graphs); **D**. Extracellular acidification rate measurements in 100 DIV dissociated organoids. The upper panel represents data from Patient 1, and the lower panel represents data from Patient 2. OCR was measured at basal levels, followed by supplementation with 1 µM oligomycin, 1 µM FCCP, and 1 µM rotenone/antimycin A; **E.** Quantification of mitochondrial respiration. Bar plots show Maximal respiration, proton leak and spear respiratory capacity. The left panel represents data from Patient 1, and the right panel represents data from Patient 2. *n* = 5 dissociated ALI-COs for heteroplasmic and control organoids, with each experiment performed in triplicate to confirm trends. Statistical analysis: one-way ANOVA with Tukey’s post hoc test (overall ANOVA *p*-values indicated in the graphs); **F.** Cytometric analysis of L1CAM immunoreactivity for neurons (left pannel) and of HEPACAM for astroglia (right panel). Graphs show also gating strategy (red and blue); **G.** Box plots showing absolute quantification of mtDNA in astrocytes sorted from 100 DIV organoids. *n* = 2 independent sorting experiments from three organoids pooled per sample. Statistical analysis: one-way ANOVA with Tukey’s post hoc test (overall ANOVA *p*-values indicated in the graphs); **H.** Heteroplasmy levels of sorted neurons and astrocytes determined by pyrosequencing. *n* = 2 independent sorting experiments from three organoids pooled per sample.

**Figure S4. A.** Venn diagram illustrating shared and unique transcription factors (TFs) identified in high, medium, and low heteroplasmy organoid groups. TF analysis was performed using SCENIC on merged data, representing transcription factors across all cell populations; Purple square emphasizes the common TFs identified in all heteroplasmic samples; **B.** Dot plots of all identified TFs in DLN5B in high, medium and low heteroplasmy; **C.** Venn diagram illustrating shared and unique transcripts for low and high DLN5B and ULN; **D.** Box plots with individual data points representing GO enrichment of major pathways in DLN5B vs. ULN for both low and high heteroplasmy. The Wilcoxon test was used to compare the distribution of continuous variables between groups, such as gene expression levels.

**Figure S5. A**. Skeletal muscle histopathology of a MELAS patient. H&E and Gomori trichrome staining (upper panels) of transverse muscle sections reveal fibre-size heterogeneity, fibrosis, rods, and fibres with internalized nuclei and cores. Muscle fibre types were distinguished by myosin ATPase staining at different pH levels, while oxidative fibres were identified by succinate dehydrogenase (SDH) activity (lower panel); **B**. Brain histopathology of a MELAS patient. H&E staining in the occipital lobe (upper panel) and cortex (lower panel) shows severe neuronal loss. GFAP immunohistochemistry reveals intense astrogliosis with widespread reactive astrocytes in MELAS versus age-matched controls. Scale bar = 100 µm; **C**. Immunofluorescence images of DIV 100 cortical organoids with high heteroplasmy and isogenic controls, showing SATB2⁺ cells (red) and TUNEL⁺ nuclei (green). Scale bar = 100 µm; **D.** Bar plots quantifying TUNEL⁺ cells co-localising with SATB2, assessing apoptosis in upper-layer neurons. Data represent the mean ± SEM; *n = 5* independent ALI-COs per heteroplasmic line and isogenic controls. Statistical analysis: one-way ANOVA with Dunnett's post hoc test (overall p-values indicated in the graphs); **E.** Immunofluorescence images of laminar organisation in DIV 70 organoid slices from high heteroplasmy and isogenic controls, with SATB2⁺ cells (red) marking ULNs and CTIP2⁺ cells (green) marking DLN5 neurons. Scale bar = 200 µm; **F.** Bar plots quantifying SATB2⁺ and CTIP2⁺ cells normalised to total cell number per ALI-CO cryosection. Data represent the mean ± SEM; *n = 3* independent ALI-COs per heteroplasmic line and isogenic controls from different batches. Statistical analysis: unpaired two-tailed t-test with Welch's correction (overall p-values indicated in the graphs); **G.** Immunofluorescence images of iPSC-derived NPCs. SOX2⁺ cells (red) mark neural stem and progenitor cells; NESTIN⁺ cells (green) label intermediate filament proteins. Scale bar = 50 µm; **H.** Bar plots quantifying SOX2⁺ and NESTIN⁺ cells normalised to total cell number. Data represent the mean ± SEM; *n = 3* independent batches. Statistical analysis: one-way ANOVA with Tukey's post hoc test (no significant differences observed); **I.** Immunofluorescence images of 2D cortical neurons positive for MAP2 (neuronal marker) and CTIP2 (deep-layer marker). *n = 3* biological replicates; **J.** Bar chart showing relative cell viability by PrestoBlue assay under basal and stressed conditions following 10 µM GSK-2837808A + 0.1% AlbuMAX for 12 hours. Data represent the mean ± SEM; *n = 6* from three independent batches. Statistical analysis: two-way ANOVA with Tukey's multiple comparisons test (no significant differences observed).

**Figure S6**. **A** and **C.** Oxygen consumption rate (OCR) measurements in day 30, 2D neurons. The upper panel represents data from Patient 1(A) and Patient 2 (C). OCR was measured at basal levels, followed by supplementation with 1 µM oligomycin, 1 µM FCCP, and 1 µM rotenone/antimycin A. Raw OCR values were normalized to total cell numbers, determined by Hoechst staining. **B** and **D.** Extracellular acidification rate measurements in day 30, 2D neurons for Patient 1 (B) AND Patient 2 (D). OCR was measured at basal levels, followed by supplementation with 1 µM oligomycin, 1 µM FCCP, and 1 µM rotenone/antimycin A; **E.** Representative immunofluorescence images of 2D control and high-heteroplasmy neurons. MAP2 (magenta) marks dendrites and cell bodies, while TOM20 (green) marks mitochondria. Scale bar: 50 µm.

**Supplemental tables**

**Table S1.** Supplementary information on the subjects related to the iPSC lines used in this study.

| **Basic information** | **Patient 1** | **Patient 2** |
| --- | --- | --- |
| Gender | Female | Female |
| Biopsy origin | Newcastle, UK | Rochester, USA |
| Race | Caucasian | - |
| Age at biopsy | 73 | 17 |
| iPSC reprograming method | Sendai virus | Sendai |
| Heteroplasmy in fibroblast | 69% | 80% |
|  |  |  |
| **Symptoms** | | |
| Stroke-like episodes | - | 36+ |
| Epilepsy | - | 36+ |
| Progressive hearing impairement | + | + |
| Diabetes | + | + |
| Pigmentary retinopathy | + | - |

**Table S2.** List of primers used, as referenced in Methods.

| **Pyrosequencing primers** | | |
| --- | --- | --- |
| Target | Primer/Probe Name | Sequence |
| A3243G | A3243G Fwd Primer | 5'-CCTCCCTGTACGAAAGGACA-3' |
|  | A3243G Rev Primer | /5'biosg/TGGCCATGGGTATGTTGTTA-3' |
|  | A3243G Sequencing | 5'-GGTTTGTTAAGATGGCAG-3' |
|  |  |  |
| **Primers for qPCR for mtDNA measurement** | | |
| Target | Primer/Probe Name | Sequence |
| B2M | B2M Fwd Primer | 5'-CGCAATCTCCAGTGACAGAA-3' |
|  | B2M Rev Primer | 5'-GCAGAATAGGCTGCTGTTCC-3' |
|  | B2M TaqMan probe | 5'-FAM/CCGTGTGAACCATGTGACTTTGTC/3'-BHQ_1 |
| MTND1 | MTND1 Fwd Primer | 5'-CAGCCGCTATTAAAGGTTCG-3' |
|  | MTND1 Rev Primer | 5'AGAGTGCGTCATATGTTGTTC-3' |
|  | MTND1TaqMan Probe | 5'-HEX/ACCCGCCACATCTACCATCACCCTC/3'-BHQ_1 |
|  |  |  |
| **Primers for ddPCR for mtDNA measurement** | | |
| Target | Primer/Probe Name | Sequence |
| ND4 | ND4 Fwd Primer | 5'-AGTGCGATGAGTAGGGGAAGG-3' |
|  | ND4 Rev Primer | 5'-ACCTTGGCTATCATCACCCGAT-3' |
|  | ND4 FAM Probe | 5'-FAM/CAACCAGCCAGAACGCCTGAACGCA/3'-BHQ_2 |
| RNAse P | RNAse P Fwd Primer | AGATTTGGACCTGCGAGCG |
|  | RNAse P Rev Primer | GAGCGGCTGTCTCCACAAGT |
|  | RNAseP HEX Probe | 5'-HEX/TTCTGACCTGAAGGCTCTGCGCG/3'-BHQ_1 |

**Table S3**. List of antibodies and their dilutions used in this study, as referenced in Methods.

| **Antibody** | **Company** | **Species** | **Catalogue** | **Application** | **Dilution** |
| --- | --- | --- | --- | --- | --- |
| ACTB | Proteintech | rabbit | Cat# 20536-1-AP | WB | 1:3000 |
| CD68 | Abcam | rabbit | Cat# ab192842 | IHC | 1:100 |
| Cleaved CASP3 | Cell Signalling | rabbit | Cat# 9664 | IF | 1:200 |
| CTIP2 | Abcam | rat | Cat# ab18465 | IF | 1:200 |
| FOXG1 | Abcam | rabbit | Cat# ab18259 | IF | 1:300 |
| GFAP | Antibodies.com | chicken | Cat# A85307 | IF | 1:1000 |
| HEPACAM | R and D Systems | mouse | Cat# MAB4108 | FACS | 1:100 |
| L1CAM | R and D Systems | mouse | Cat# MAB7771 | FACS | 1:100 |
| MAP2 | Antibodies.com | chicken | Cat# A85363 | IF | 1:1000 |
| MTATP6 | Cell Signalling | rabbit | Cat# 70262S | WB | 1:2000 |
| MTCO1 | Abcam | mouse | Cat# ab14705 | WB | 1:1000 |
| MTCO2 | Abcam | mouse | Cat# ab110258 | WB | 1:1000 |
| NEST | Sigma-Aldrich | rabbit | Cat# N5413 | IF | 1:300 |
| NeuN | Sigma-Aldrich | rabbit | Cat# ABN78 | IF | 1:500 |
| SATB2 | Abcam | mouse | Cat# ab51502 | IF | 1:300 |
| SMI-321 | BioLegend | mouse | Cat# 837904 | IF | 1:200 |
| SOX2 | R and D Systems | goat | Cat# AF2018 | IF | 1:200 |
| TOM20 | Abcam | rabbit | Cat# ab186735 | WB | 1:2000 |
| Total OXPHOS Human WB Antibody Coctail Kit | Abcam | mouse | Cat# ab110411 | WB | 1:800 |
| TUJ | Abcam | mouse | Cat# ab78078 | IF | 1:500 |
| UQCRC1 | Abcam | mouse | Cat# ab110252 | WB | 1:2000 |
| Donkey Anti Goat IgG(H+L) Highly Crossed Absorbed Secondary Antibody, Alexa Fluor Plus 647 | Thermo Fisher Scientific |  | Cat# A32849TR | IF | 1:500 |
| Donkey Anti Mouse IgG(H+L) Highly Crossed Absorbed Secondary Antibody, Alexa Fluor 568 | Thermo Fisher Scientific |  | Cat# A10037 | IF | 1:500 |
| Donkey Anti Rabbit IgG(H+L) Highly-Cross Absorbed Secondary Antibody, Alexa Fluor 488 | Thermo Fisher Scientific |  | Cat# A-21206 | IF | 1:500 |
| Donkey Anti Rabbit IgG(H+L) Highly-Cross Absorbed Secondary Antibody, Alexa Fluor 568 |  |  | Cat# SA5-10042 | IF | 1:500 |
| Donkey Anti Rat IgG H&L Alexa Fluor 647 preabsorbed | Abcam |  | Cat# ab150155 | IF | 1:500 |
| Goat Anti Chicken IgG(H+L) Cross-Absorbed Secondary Antibody Alexa Flour 488 | Thermo Fisher Scientific |  | Cat# A-11006 | IF | 1:500 |
| Goat Anti Chicken IgY(H+L) Cross-Absorbed Secondary Antibody Alexa Flour 647 | Thermo Fisher Scientific |  | Cat# A48260 | IF | 1:500 |
| Goat Anti Mouse IgG (H+L) Secondary Antibody, HRP | Thermo Fisher Scientific |  | Cat# G-21040 | WB | 1:5000 |
| Goat Anti Rabbit IgG (H+L) Secondary Antibody, HRP | Thermo Fisher Scientific |  | Cat# 31462 | WB | 1:5000 |
